# Characterizing the US Population by Patterns of Mobile Health Use for Health and Behavioral Tracking: Analysis of the National Cancer Institute's Health Information National Trends Survey Data

**DOI:** 10.2196/16299

**Published:** 2020-05-14

**Authors:** Camella J Rising, Roxanne E Jensen, Richard P Moser, April Oh

**Affiliations:** 1 Health Communication and Informatics Research Branch, Behavioral Research Program Division of Cancer Control and Population Sciences National Cancer Institute Rockville, MD United States; 2 Outcomes Research Branch, Healthcare Delivery Research Program Division of Cancer Control and Population Sciences National Cancer Institute Rockville, MD United States; 3 Office of the Associate Director, Behavioral Research Program Division of Cancer Control and Population Sciences National Cancer Institute Rockville, MD United States

**Keywords:** mobile health, population health, health communication, survey methodology, mobile applications, devices

## Abstract

**Background:**

Multiple types of mobile health (mHealth) technologies are available, such as smartphone health apps, fitness trackers, and digital medical devices. However, despite their availability, some individuals do not own, do not realize they own, or own but do not use these technologies. Others may use mHealth devices, but their use varies in tracking health, behaviors, and goals. Examining patterns of mHealth use at the population level can advance our understanding of technology use for health and behavioral tracking. Moreover, investigating sociodemographic and health-related correlates of these patterns can provide direction to researchers about how to target mHealth interventions for diverse audiences.

**Objective:**

The aim of this study was to identify patterns of mHealth use for health and behavioral tracking in the US adult population and to characterize the population according to those patterns.

**Methods:**

We combined data from the 2017 and 2018 National Cancer Institute Health Information National Trends Survey (N=6789) to characterize respondents according to 5 mutually exclusive reported patterns of mHealth use for health and behavioral tracking: (1) mHealth nonowners and nonusers report not owning or using devices to track health, behaviors, or goals; (2) supertrackers track health or behaviors and goals using a smartphone or tablet plus other devices (eg, Fitbit); (3) app trackers use only a smartphone or tablet; (4) device trackers use only nonsmartphone or nontablet devices and do not track goals; and (5) nontrackers report having smartphone or tablet health apps but do not track health, behaviors, or goals.

**Results:**

Being in the mHealth nonowners and nonusers category (vs all mHealth owners and users) is associated with males, older age, lower income, and not being a health information seeker. Among mHealth owners and users, characteristics of device trackers and supertrackers were most distinctive. Compared with supertrackers, device trackers have higher odds of being male (odds ratio [OR] 2.22, 95% CI 1.55-3.19), older age (vs 18-34 years; 50-64 years: OR 2.83, 95% CI 1.52-5.30; 65+ years: OR 6.28, 95% CI 3.35-11.79), have an annual household income of US $20,000 to US $49,999 (vs US $75,000+: OR 2.31, 95% CI 1.36-3.91), and have a chronic condition (OR 1.69, 95% CI 1.14-2.49). Device trackers also have higher odds of not being health information seekers than supertrackers (OR 2.98, 95% CI 1.66-5.33).

**Conclusions:**

Findings revealed distinctive sociodemographic and health-related characteristics of the population by pattern of mHealth use, with notable contrasts between those who do and do not use devices to track goals. Several characteristics of individuals who track health or behaviors but not goals (device trackers) are similar to those of mHealth nonowners and nonusers. Our results suggest patterns of mHealth use may inform how to target mHealth interventions to enhance reach and facilitate healthy behaviors.

## Introduction

### Background

Mobile health (mHealth) includes the use of portable digital devices, such as smartphones, tablet computers, and fitness and medical wearables, to support health. Approximately 80% of US adults own a smartphone [1], which typically has one or more health-related software apps (*health apps*) preinstalled at the point of purchase. Close to 60% of smartphone owners also report installing (ie, downloading) one or more health apps onto their smartphone [2]. Wearable devices with a multitude of sensor types designed to monitor health indicators, such as activity, sleep, blood glucose, and blood pressure, have also penetrated the US marketplace [3,4]. Within the general population, wearable activity monitors (eg, Fitbit) are among the most commonly owned wearable devices and are reportedly used by about 13% of Americans [5].

Despite their ubiquity, ownership and use of diverse types of mHealth technologies for health and behavioral tracking varies. Reported use of mHealth technologies includes communicating with health care providers [6], making dietary or physical activity decisions [7,8], achieving health goals such as weight loss [9], and monitoring chronic conditions such as diabetes or hypertension [10,11]. Some mHealth intervention studies have reported that the use of mHealth technologies is associated with improved health or behavioral outcomes. These include, for example, improvements in diet and physical activity [12,13], weight loss or maintenance and blood glucose reduction [11], and higher quality patient-provider communication [14]. Although these studies suggest the potential for mHealth to enhance patient-provider communication, improve health outcomes, and facilitate behavior change, research examining how mHealth technologies are used by the US population for health and behavioral tracking is needed to understand how to target and tailor mHealth interventions to enhance their reach and efficacy across population subgroups.

Currently, there are gaps in the literature related to understanding use of mHealth technologies for health and behavioral tracking that need to be filled to advance mHealth intervention science. First, most intervention studies to date evaluate only one type of mHealth technology to address a desired health outcome or target behavior [8,15-18]. Yet, as mHealth technologies diversify in functionality, individuals have also become increasingly diverse in the types of mHealth technologies they own and use, with multiuse becoming more common [19]. Therefore, identifying the types of mHealth technologies the US population uses for health or behavioral tracking is an essential component of advancing the science.

Second, there is limited research about whether people in the United States set health goals when they use mHealth technologies for health or behavioral tracking. Goal setting can promote higher and sustained engagement with mHealth interventions [20-22]. Moreover, goal setting is strongly associated with favorable health behavior outcomes, such as increased physical activity and healthy eating in overweight and obese adults [23] and is a characteristic of efficacious mHealth interventions to improve diet, physical activity, and sedentary behaviors of children and adults in the general population [13]. Investigating whether tracking includes goal setting and assessing the characteristics of those who set these goals may increase understanding about who may be more (or less) likely to change health behavior as a result of using mHealth technologies for health or behavioral tracking and facilitate more precise targeting and tailoring of future mHealth interventions.

Finally, past studies have found that many people in the United States report not owning or using mHealth technologies for health or behavioral tracking, and that these individuals differ from mHealth technology owners and users on several characteristics such as age, gender, education, and income [5,24]. However, mHealth technologies for health and behavioral tracking continue to evolve and are adopted at different rates across population subgroups. Thus, continuing to describe and characterize mHealth nonowners and nonusers is necessary to understand patterns of mHealth use for health and behavioral tracking in the US population.

### Study Aims

This paper has 3 primary aims. The first is to address gaps in the literature by describing patterns of mHealth use for health and behavioral tracking in the US population. We account for 3 factors in our conceptualization of pattern of mHealth use: (1) whether mHealth technologies are owned and used for health or behavioral tracking, (2) the types of mHealth technologies owned and used for health or behavioral tracking, namely smartphones or tablets and other digital devices such as fitness trackers or medical devices, and (3) whether health or behavioral tracking with mHealth technologies includes goal setting.

On the basis of these 3 factors, we distinguish between 5 mutually exclusive categories, or population subgroups, of mHealth owners and users and mHealth nonowners and nonusers in the United States. *mHealth nonowners and nonusers* are those who report that they do not own or use mHealth technologies for health or behavioral tracking. Among mHealth owners and users, *supertrackers* are those who report using multiple devices—a smartphone or tablet and another device, such as a fitness tracker or medical device—to track health or behaviors and goals, whereas *app trackers* report only using a smartphone or tablet for the same purpose. *Device trackers* are those who report only using a device other than a smartphone or tablet to track health or behaviors and do not use smartphone or tablet health apps to track goals. Finally, *nontrackers* are those who report only having smartphone or tablet health apps but do not use them to track goals and do not track health or behaviors with other devices.

The second aim of this paper is to describe the characteristics of mHealth nonowners and nonusers by comparing them with people who own or use mHealth technologies for health and behavioral tracking. We consider individual-level factors such as sociodemographics—age, gender, race and ethnicity, education, income, and geographical area. We also consider factors associated with health and health behaviors, such as weight status, having a chronic condition (eg, diabetes), perceived health status, health self-efficacy, and being a health information seeker.

The third and final aim of this paper is to describe and compare the characteristics (sociodemographics, health, and health behaviors) of mHealth owners and users by pattern of mHealth use for health and behavioral tracking. Comparing mHealth owners and users by pattern of mHealth use may provide important insights about those who not only own and use different types of mHealth technologies in the United States but also use those technologies in ways that can promote improved health and health behavior outcomes (ie, goal setting). As mHealth technology development and adoption rapidly evolves, characterizing the population by pattern of mHealth use may inform how to target and tailor mHealth interventions to reach diverse US audiences and facilitate healthy behaviors.

## Methods

### Data

Data were merged from the National Cancer Institute (NCI)’s Health Information National Trends Survey (HINTS), HINTS 5, Cycle 1, and HINTS 5, Cycle 2. HINTS is a probability-based, cross-sectional survey of the US adult, civilian, noninstitutionalized population. Survey data were collected by paper-and-pencil self-administered questionnaires completed from January through May 2017 (Cycle 1; see Multimedia Appendix 1) and January through May 2018 (Cycle 2, see Multimedia Appendix 2). HINTS has a two-stage, stratified sample; addresses were randomly selected from a US Postal Service file of residential addresses, and a random individual respondent was selected from each sample household (HINTS 5, Cycle 1 response rate=32.4%, N=3285; HINTS 5, Cycle 2 response rate=32.9%, N=3504). Details related to HINTS methodology have been described elsewhere [25].

### Sociodemographic and Health-Related Characteristics

Items included in the analysis pertaining to respondent characteristics were asked of the full, merged sample from 2017 and 2018 (N=6789). Sociodemographic variables included in analyses were gender (male and female); age (18-34, 35-49, 50-64, and ≥65 years old); race (white, black, and other race, which combines low-frequency responses for American Indian/Alaska Native, Asian Indian, Chinese, Filipino, Japanese, Korean, Vietnamese, other Asian, Native Hawaiian, Guamanian or Chamorro, Samoan, and other Pacific Islander); Hispanic ethnicity (Hispanic, non-Hispanic); education (less than high school or high school graduate, technical, vocational, or some college, and college graduate or postgraduate); annual household income (<US $20,000, US $20,000-US $49,999, US $50,000-US $74,999, and ≥US $75,000); and geographical area. Geographical area was categorized as urban or rural based on the US Department of Agriculture Economic Research Service continuum codes.

Health-related variables included BMI (normal: 18.5-24.9; overweight: 25-29.9; and obese: ≥30), perceived health status (*poor* or *fair*, *good*, *very good*, and *excellent*), health self-efficacy (*not confident at all* or *a little confident*, *somewhat confident*, *very confident*, and *completely confident*), having one or more chronic conditions (diabetes, high blood pressure, or a heart condition), and being a health information seeker. Perceived health status was evaluated with a single item, *In general, would you say your health is…* This single item includes a 5-point Likert response from *poor* to *excellent*. Health self-efficacy was measured with the item, *Overall, how confident are you about your ability to take good care of your health?* Response options were also on a 5-point Likert scale, ranging from *completely confident* to *not confident at all*. Having one or more chronic conditions was reported using a checklist that followed the question, *Has a doctor or other health professional ever told you that you had any of the following medical conditions?* In this study, respondents were classified as having a chronic condition if they selected *yes* to *diabetes or high blood sugar*, *high blood pressure or hypertension*, or *a heart condition such as heart attack, angina, or congestive heart failure*. These chronic conditions were included because self-management using digital medical devices (eg, glucometer) is common. The item, *Have you ever looked for information about health or medical topics from any source?* (*yes* or *no*), was used to evaluate being a health information seeker.

#### Mobile Health Technologies Owned and Used for Health or Behavioral Tracking and Goal Setting

Respondents with a smartphone or tablet computer were asked, *On your tablet or smartphone, do you have any apps related to health and wellness?* (*yes*, *no*, or *don’t know*). For logistic regression analyses, this response was dichotomized to yes vs no and don’t know. *Don’t know* responses were collapsed with *no* responses owing to a low response frequency for *don’t know* (n=285) and because not knowing whether one has health apps suggests nonuse of those apps for health or behavioral tracking. Respondents were also asked, *Has your tablet or smartphone helped you track progress on a health-related goal such as quitting smoking, losing weight, or increasing physical activity?* (*yes* or *no*). All respondents were asked, *Other than a tablet or smartphone, have you used an electronic device to monitor or track your health within the last 12 months? Examples include Fitbit, blood glucose meters, and blood pressure monitors*. (*yes* or *no*).

#### Operationalizing Pattern of Mobile Health Use for Health and Behavioral Tracking

**Figure 1:**
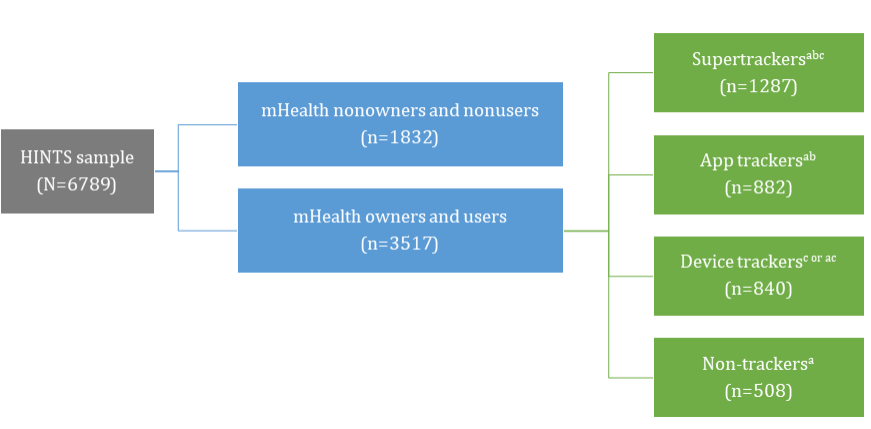
Operationalizing pattern of mobile health use for health and behavioral tracking. a. On your tablet or smartphone, do you have any “apps” related to health and wellness? Yes. b. Has your tablet or smartphone helped you track progress on a health-related goal such as quitting smoking, losing weight, or increasing physical activity? Yes. c. Other than a tablet or smartphone, have you used an electronic device to monitor or track your health within the last 12 months? Examples include Fitbit, blood glucose meters, and blood pressure monitors. Yes. HINTS: Health Information National Trends Survey.

Figure 1 represents our operationalization of each mutually exclusive pattern of mHealth use for health and behavioral tracking. The combined HINTS sample was divided into mHealth owners and users and nonowners and nonusers. mHealth nonowners and nonusers were defined as those who reported that they do not have or do not know if they have health apps on their smartphone or tablet, do not use a smartphone or tablet to track health-related goals, and do not use other devices to track health or behaviors (eg, Fitbit, glucose meter, and blood pressure monitor). mHealth owners and users were divided into 4 categories based on distinctive ways mHealth technologies are owned or used to track health or behaviors and goals. Supertrackers were defined as those who reported using a smartphone or tablet to track health-related goals and use other devices, such as a fitness tracker or digital medical device, to track health or behaviors. App trackers were defined as those who reported using a smartphone or tablet to track health-related goals but do not use other devices to track health or behaviors. Device trackers were defined as those who reported using devices other than a smartphone or tablet to track health or behaviors but do not use a smartphone or tablet to track health-related goals. Finally, nontrackers were defined as those who only reported having smartphone or tablet health apps but do not use them to track health-related goals and do not use other devices to track health or behaviors.

### Statistical Analysis

Analyses of merged HINTS 5, Cycle 1 and HINTS 5, Cycle 2 data were conducted using SAS version 9.4 (SAS Institute, Cary, NC). Descriptive statistics (frequencies, weighted percentages, and chi-square statistics) were used to examine patterns of mHealth use for health and behavioral tracking (Aim 1). Respondents with and without a smartphone or tablet were included in the mHealth nonowners and nonusers category; not having, not tracking, and not monitoring health with a mobile device was the primary criterion for inclusion. Similar to mHealth nonowners and nonusers, having a smartphone or tablet was not a criterion for inclusion in the device tracker category.

In all, 2 logistic regression models were constructed to characterize and compare US population subgroups by pattern of mHealth use for health and behavioral tracking. To address Aim 2, binomial logistic regression was performed to test the odds that respondent characteristics independently predict being in the mHealth nonowners and nonusers category, while holding the other respondent characteristics constant (reference category=all mHealth owners and users). To address Aim 3, a multinomial logistic regression model using the generalized logit was constructed to determine the predicted probability that respondent characteristics independently predict mHealth owners’ and users’ pattern of mHealth use for health and behavioral tracking, while controlling for the other respondent characteristics. Because a normative group of mHealth owners and users does not exist, supertrackers were chosen as the reference category for this second model as Aim 1 findings showed supertrackers made up the largest category of mHealth owners and users in the sample. Tests for significance for odds ratios and 95% CI were calculated at *P*<.05. Complete case analysis and listwise deletion were used for regression models.

Data weights were applied to provide representative US population estimates. A full-sample weight was used to calculate population-level point estimates and parameters. Replicate weights (calculated using the jackknife variance estimation method) were used to compute accurate standard errors.

## Results

### Sociodemographic and Health-Related Characteristics

The total combined HINTS sample was 6789. Weighted prevalence estimates were calculated for HINTS respondents’ sociodemographic and health-related characteristics (see Multimedia Appendix 3). HINTS poststratification weightings adjust for inherent variations in the obtained sample by adjusting weighted totals to approximate known population characteristics. As in most health surveys, the HINTS raw sample tended to overaccumulate responses from females, older age individuals, people who are urban-dwelling, and those with more education and higher annual household income. In addition, white, non-Hispanic respondents comprised more than half of the sample. With respect to health-related characteristics, about half of the US adult population reported at least very good perceived health status, and most were at least very confident about their health self-efficacy. Most were health information seekers, overweight or obese, and had never been diagnosed with diabetes, hypertension, or a heart condition.

### Mobile Health Technologies Owned and Used for Health or Behavioral Tracking and Goal Setting

Weighted prevalence estimates were also calculated for items pertaining to ownership and use of mHealth technologies for health or behavioral tracking and goal setting. Most respondents (85.22%) reported having a smartphone or tablet, close to half (46.83%) reported having a health app on one of these devices, and 43.23% reported that they use their smartphone or tablet to track progress on a health-related goal. Approximately one-third of respondents (34.66%) reported that they have a device other than a smartphone or tablet to track their health or behaviors.

### Aim 1: Patterns of Mobile Health Use for Health and Behavioral Tracking

On the basis of our operationalization of pattern of mHealth use for health and behavioral tracking, mHealth nonowners and nonusers made up about one-third of the sample (34.72%). Most mHealth nonowners and nonusers were owners of a smartphone or tablet (95.15%). Nontrackers, those who reported having health apps on their smartphone or tablet but do not use them to track a health-related goal and do not use other devices to track health or behaviors, comprised the smallest percentage of the sample (8.8%).

Among respondents who reported owning or using mHealth technologies for health or behavioral tracking, supertrackers were the most prevalent (24.05%), followed by app trackers (19.5%) and device trackers (12.9%). Most device trackers reported having a smartphone or tablet (97.6%), and approximately one-third reported that they have a health app on one of those devices (35.0%). Table 1 describes the sociodemographic and health-related characteristics of respondents by pattern of mHealth use for health and behavioral tracking.

**Table 1:** Weighted population estimates for sociodemographic and health-related characteristics by pattern of mobile health use for health and behavioral tracking; HINTS 5, Cycle 1, 2017 and Cycle 2, 2018 (N=6789).

Characteristics		Nonowners and nonusers (n=1832; 34.72%)	Nontrackers (n=508; 8.8%)	Device trackers (n=840; 12.9%)	App trackers (n=882; 19.5%)	Supertrackers (n=1287; 24.05%)	Chi-square (*df*)	*P* value
**Gender, n (%)**							21.6 (4)	<.001
	Male	733 (51.90)	192 (46.4)	381 (58.0)	298 (46.2)	445 (41.93)	N/A^a^	N/A
	Female	955 (48.10)	290 (53.6)	396 (42.0)	542 (53.8)	776 (58.07)	N/A	N/A
**Age (years), n (%)**							222.5 (12)	<.001
	18-34	180 (21.25)	61 (22.6)	42 (12.0)	244 (42.1)	216 (27.56)	N/A	N/A
	35-49	355 (28.47)	117 (29.4)	96 (22.1)	267 (33.1)	390 (33.67)	N/A	N/A
	50-64	645 (33.36)	183 (33.5)	289 (37.9)	255 (19.9)	458 (30.76)	N/A	N/A
	≥65	583 (16.92)	133 (14.5)	388 (28.0)	101 (4.9)	203 (8.01)	N/A	N/A
**Race and ethnicity, n (%)**							29.3 (12)	.004
	White, non-Hispanic	1,046 (64.50)	315 (70.7)	538 (75.0)	487 (60.9)	756 (63.92)	N/A	N/A
	Black, non-Hispanic	204 (9.91)	60 (10.5)	87 (6.9)	130 (10.5)	182 (11.60)	N/A	N/A
	Hispanic	272 (17.72)	52 (12.9)	83 (11.0)	148 (19.1)	155 (13.69)	N/A	N/A
	Other	142 (7.87)	45 (5.9)	62 (7.1)	72 (9.5)	123 (10.79)	N/A	N/A
**Education, n (%)**							109.9 (8)	<.001
	High school graduate or less	514 (35.42)	84 (28.5)	170 (26.3)	150 (21.2)	148 (16.37)	N/A	N/A
	Technical, vocational, or some college	566 (38.31)	150 (30.9)	284 (43.2)	259 (38.5)	337 (36.84)	N/A	N/A
	College graduate or postgraduate	705 (26.27)	266 (40.6)	369 (30.5)	466 (40.3)	791 (46.79)	N/A	N/A
**Income in US $, n (%)**							106.5 (12)	<.001
	<20,000	360 (19.04)	45 (9.4)	109 (12.0)	125 (14.9)	104 (10.23)	N/A	N/A
	20,000-49,999	530 (29.04)	111 (18.6)	242 (27.4)	223 (27.7)	214 (14.91)	N/A	N/A
	50,000-74,999	342 (20.76)	100 (19.1)	159 (19.4)	160 (16.9)	243 (19.93)	N/A	N/A
	≥75,000	566 (31.16)	245 (52.9)	316 (41.2)	367 (40.5)	712 (54.93)	N/A	N/A
**Geographical area, n (%)**							15.4 (4)	.004
	Urban	1569 (85.83)	460 (90.2)	709 (82.4)	782 (87.8)	1,170 (90.39)	N/A	N/A
	Rural	263 (14.17)	48 (9.8)	131 (17.6)	100 (12.2)	117 (9.61)	N/A	N/A
**Perceived health status, n (%)**							47.4 (12)	<.001
	Poor or fair	281 (14.70)	58 (8.2)	179 (21.5)	102 (11.7)	141 (9.89)	N/A	N/A
	Good	643 (35.14)	170 (36.5)	315 (38.8)	257 (31.2)	399 (30.42)	N/A	N/A
	Very good	672 (36.92)	206 (41.9)	267 (30.7)	371 (37.4)	551 (45.23)	N/A	N/A
	Excellent	219 (13.24)	70 (13.4)	70 (9.0)	145 (19.7)	189 (14.46)	N/A	N/A
**Health self-efficacy, n (%)**							19.4 (12)	.08
	A little confident or not confident at all	89 (5.11)	15 (2.7)	51 (8.3)	22 (3.2)	35 (3.50)	N/A	N/A
	Somewhat confident	442 (26.83)	112 (25.5)	221 (26.3)	174 (20.1)	276 (23.31)	N/A	N/A
	Very confident	845 (44.51)	237 (46.5)	383 (45.2)	412 (44.9)	634 (48.53)	N/A	N/A
	Completely confident	440 (23.55)	138 (25.3)	178 (20.2)	268 (31.8)	337 (24.66)	N/A	N/A
**Health information seeker, n (%)**							66.15 (4)	<.001
	Yes	1,369 (76.76)	438 (84.2)	712 (82.8)	757 (85.1)	1,191 (92.43)	N/A	N/A
	No	439 (23.24)	59 (15.8)	118 (17.2)	118 (14.9)	84 (7.57)	N/A	N/A
**BMI, n (%)**							19.5 (8)	.01
	Normal (18.5-24.9)	565 (33.11)	195 (41.1)	202 (26.6)	286 (35.1)	344 (28.19)	N/A	N/A
	Overweight (25-29.9)	610 (33.61)	152 (30.2)	298 (31.9)	301 (34.6)	451 (36.19)	N/A	N/A
	Obese (≥30)	561 (33.28)	145 (28.7)	304 (41.5)	269 (30.3)	452 (35.62)	N/A	N/A
**One or more chronic conditions, n (%)**							104.0 (4)	<.001
	Yes	848 (35.71)	213 (34.6)	609 (65.6)	277 (26.5)	594 (39.09)	N/A	N/A
	No	984 (64.29)	295 (65.4)	231 (34.4)	605 (73.5)	693 (60.91)	N/A	N/A

^a^N/A: not applicable.

### Aim 2: Characteristics Associated With Being in the Mobile Health Nonowners and Nonusers Category

Compared with mHealth owners and users (nontrackers, app trackers, device trackers, and supertrackers), mHealth nonowners and nonusers had higher odds of being male (OR 1.38, 95% CI 1.08-1.77) and 35 years old or older, with the odds of being in the mHealth nonowners and nonusers category increasing with advancing age (35-49 years old: OR 1.55, 95% CI 1.01-2.36; 50-64 years old: OR 2.01, 95% CI 1.30-3.11; 65+ years old: OR 2.77, 95% CI 1.86-4.13). mHealth nonowners and nonusers also had higher odds of reporting an annual household income below US $75,000 (<US $20,000: OR 2.06, 95% CI 1.34-3.16; US $20,000-US $49,999: OR 1.94, 95% CI 1.33-2.83; US $50,000-US $74,999: OR 1.67, 95% CI 1.14-2.46).

In addition, mHealth nonowners and nonusers had higher odds of not being a college graduate (high school graduate or less: OR 1.88, 95% CI 1.31-2.69; technical, vocational, or some college: OR 1.36, 95% CI 1.02-1.83) and not being health information seekers (OR 1.53, 95% CI 1.15-2.04). However, they had lower odds of reporting a chronic condition (OR 0.57, 95% CI 0.45-0.72) compared with mHealth owners and users. There were nonsignificant differences for race and ethnicity (*F*_3,98_=0.11; *P*=.95), geographical area (*F*_1,98_=0.39; *P*=.54), perceived health status (*F*_3,98_=0.31; *P*=.82), health self-efficacy (*F*_3,98_=0.92; *P*=.43), and weight status (BMI, *F*_1,98_=1.77; *P*=.18) when controlling all other variables (see Table 2).

**Table 2:** Sociodemographic and health-related characteristics significantly associated with being in the mobile health nonowners and nonusers category (n=1468). Reference category=all mobile health owners and users.

Characteristic		Odds ratio (95% CI)		*P* value	
**Gender**					
	Female		Reference		N/A^a^
	Male		1.38 (1.08-1.77)		.01
**Age (years)**					
	18-34		Reference		N/A
	35-49		1.55 (1.01-2.36)		.04
	50-64		2.01 (1.30-3.11)		.002
	≥65		2.77 (1.86-4.13)		<.001
**Education**					
	College graduate or postgraduate		Reference		N/A
	Technical, vocational, or some college		1.36 (1.02-1.83)		.04
	High school graduate or less		1.88 (1.31-2.69)		.001
**Income in US $**					
	≥75,000		Reference		N/A
	50,000-74,999		1.67 (1.14-2.46)		.009
	20,000-49,999		1.94 (1.33-2.83)		<.001
	<20,000		2.06 (1.34-3.16)		.001
**Health information seeker**					
	Yes		Reference		N/A
	No		1.53 (1.15-2.04)		.004
**One or more chronic conditions**					
	No		Reference		N/A
	Yes		0.57^b^ (0.45-0.72)		<.001

^a^N/A: not applicable.

^b^Indicates a negative association.

### Aim 3: Characteristics Associated With Mobile Health Owners’ and Users’ Pattern of Mobile Health Use for Health and Behavioral Tracking

Multinomial logistic regression analysis revealed several sociodemographic and health-related characteristics associated with pattern of mHealth use for health and behavioral tracking among mHealth owners and users (reference category=supertrackers). As shown in Table 3, there were significant differences in terms of gender, age, race and ethnicity, income, having a chronic condition, weight status, and being a health information seeker. There were, however, nonsignificant differences for pattern of mHealth use by geographical area (*F*_3,98_=1.37; *P*=.29), perceived health status (*F*_9,98_=1.33; *P*=.23), health self-efficacy (*F*_9,98_=0.91; *P*=.52), and education (*F*_6,98_=1.47; *P*=.20) when controlling for all other variables.

Compared with supertrackers, the largest category of mHealth owners and users in this study, app trackers had higher odds of being male (OR 1.46, 95% CI 1.11-1.94) and lower odds of being 50 years old or older (50-64 years old: OR 0.63, 95% CI 0.41-0.96; 65+ years old: OR 0.50, 95% CI 0.29-0.88). App trackers had about 2 times the odds of reporting an annual household income in the US $20,000-US $49,999 range than US $75,000 or more (OR 2.32, 95% CI 1.61-3.33). Finally, app trackers had lower odds of reporting that they have a chronic condition than supertrackers (OR 0.62, 95% CI 0.42-0.92).

Device trackers also had higher odds of being male (OR 2.22, 95% CI 1.55-3.19) compared with supertrackers. With respect to age, device trackers had nearly 3 times the odds of being 50-64 years old (OR 2.83, 95% CI 1.52-5.30) and about 6 times the odds of being 65 years old or older (OR 6.28, 95% CI 3.35-11.79). Device trackers had about 2 times the odds of reporting an annual household income in the US $20,000-US $49,999 range than US $75,000 or more when compared with supertrackers (OR 2.31, 95% CI 1.36-3.91) and about 3 times the odds of not being health information seekers (OR 2.98, 95% CI 1.66-5.33). Device trackers had lower odds of identifying as non-Hispanic black (OR 0.48, 95% CI 0.31-0.74) and other race and ethnicity (OR 0.54, 95% CI 0.30-0.99). They had higher odds of reporting a chronic condition (OR 1.69, 95% CI 1.14-2.49) but lower odds of being overweight (OR 0.58, 95% CI 0.38-0.88) or obese (OR 0.55, 95% CI 0.35-0.86) than supertrackers.

Finally, compared with supertrackers, nontrackers had more than twice the odds of being 65 years old or older (OR 2.74, 95% CI 1.47-5.11). They also had more than 2 times the odds of not being health information seekers (OR 2.37, 95% CI 1.16-4.86). Nontrackers had lower odds of being in the other race and ethnicity category (OR 0.51, 95% CI 0.27-0.96) than supertrackers. In addition, they had lower odds of being overweight (OR 0.47, 95% CI 0.29-0.76) or obese (OR 0.43, 95% CI 0.28-0.67).

**Table 3:** Characteristics significantly associated with being a mobile health (mHealth) owner and user by pattern of mHealth use for health and behavioral tracking. Reference category=Supertrackers (n=1122).

Characteristic		App trackers (n=773), OR (95% CI)	*P* value	Device trackers (n=685), OR (95% CI)	*P* value	Nontrackers (n=428), OR (95% CI)	*P* value
**Gender**							
	Female (ref)	N/A^a^	N/A	N/A	N/A	N/A	N/A
	Male	1.46 (1.11-1.94)	.008	2.22 (1.55-3.19)	<.001	1.47 (0.96-2.25)	.07
**Age (years)**							
	18-34 (ref)	N/A	N/A	N/A	N/A	N/A	N/A
	35-49	0.80 ^b^ (0.54-1.19)	.27	1.41 (0.70-2.85)	.34	1.29 (0.70-2.38)	.42
	50-64	0.63 ^b^ (0.41-0.96)	.03	2.83 (1.52-5.30)	.001	1.65 (0.84-3.24)	.14
	>65	0.50^b^ (0.29-0.88)	.02	6.28 (3.35-11.79)	<.001	2.74 (1.47-5.11)	.002
**Race and ethnicity**							
	White, non-Hispanic (ref)	N/A	N/A	N/A	N/A	N/A	N/A
	Black, non-Hispanic	1.02 (0.65-1.62)	.93	0.48^b^ (0.31-0.74)	.001	0.95^b^ (0.51-1.75)	.86
	Hispanic	1.23 (0.80-1.88)	.35	0.75^b^ (0.44-1.29)	.29	0.82^b^ (0.42-1.60)	.56
	Other	0.92^b^ (0.47-1.81)	.81	0.54^b^ (0.30-0.99)	.046	0.51^b^ (0.27-0.96)	.04
**Income in US $**							
	>75,000 (ref)	N/A	N/A	N/A	N/A	N/A	N/A
	50,000-74,999	1.22 (0.78-1.92)	.39	1.31 (0.81-2.12)	.27	1.10 (0.66-1.82)	.71
	20,000-49,999	2.32 (1.61-3.33)	<.001	2.31 (1.36-3.91)	.002	1.25 (0.71-2.20)	.44
	<20,000	1.76 (0.83-3.73)	.14	1.33 (0.67-2.65)	.41	0.87^b^ (0.33-2.31)	.78
**Health information seeker**							
	Yes (ref)	N/A	N/A	N/A	N/A	N/A	N/A
	No	1.70 (0.86-3.35)	.12	2.98 (1.66-5.33)	<.001	2.37 (1.16-4.86)	.02
**BMI (kg/m^2^)**							
	Normal (18.5-24.9; ref)	N/A	N/A	N/A	N/A	N/A	N/A
	Overweight (25-29.9)	0.87^b^ (0.57-1.33)	.51	0.58^b^ (0.38-0.88)	.01	0.47^b^ (0.29-0.76)	.002
	Obese (≥30)	0.71^b^ (0.45-1.14)	.15	0.55^b^ (0.35-0.86)	.009	0.43^b^ (0.28-0.67)	<.001
**One or more chronic conditions**							
	No (ref)	N/A	N/A	N/A	N/A	N/A	N/A
	Yes	0.62^b^ (0.42-0.92)	.02	1.69 (1.14-2.49)	.009	0.67^b^ (0.44-1.02)	.06

^a^N/A: not applicable.

^b^Indicates a negative association.

## Discussion

### Principal Findings

The aims of this study were to examine patterns of mHealth use for health and behavioral tracking in the US population and to characterize the population according to those patterns. We found that those who do not own or use mHealth technologies for health or behavioral tracking make up a relatively large proportion of the population (about one-third). Among owners and users of mHealth technologies, being a supertracker—using multiple mHealth technologies for health or behavioral tracking and goal setting—is the most common pattern of mHealth use. Our study also confirms and extends what other studies of nationally representative samples (eg, Canada) have discovered [7]; that is, there are substantive differences among mHealth technology owners and users in terms of sociodemographics and health-related factors. The sociodemographic and health-related characteristics of supertrackers and device trackers were the most distinctive, with several characteristics of device trackers—those who track health or behaviors but not health goals—paralleling those of mHealth nonowners and nonusers. Our findings suggest that the distinctive characteristics of supertrackers and device trackers, in particular, can be used to help target and tailor future mHealth interventions.

#### Characteristics of Nonowners and Nonusers of Mobile Health Technologies for Health and Behavioral Tracking

People who do not own or use mHealth technologies for health and behavioral tracking make up about one-third of the US population; regardless of the high percentage of Americans who own a smartphone or tablet, ownership does not necessarily mean that these devices are being used to track health, behaviors, or goals. We found that mHealth nonowners and nonusers tend to be male, older age, and report lower education and income levels than owners and users of mHealth technologies for health or behavioral tracking, which are findings supported by other studies focused on the US population’s mHealth technology usage [5,24]. In addition, our findings revealed a relationship between being in the mHealth nonowners and nonusers category and not seeking health information in general.

mHealth nonowners and nonusers also tended not to have a chronic condition, specifically diabetes, hypertension, or a heart condition, when compared with owners and users of mHealth technologies for health or behavioral tracking. These results suggest that having a chronic condition is a potential reason or motivator for owning and using mHealth technologies. Although a study of smartphone owners found that those with chronic conditions are no more likely to use health apps than people without a chronic condition [10], our analyses also accounted for respondents’ use of other devices, such as Fitbits, glucometers, and blood pressure monitors. Thus, our analyses appear to have detected distinctive differences in use of mHealth technologies between those with and without a chronic condition because these diverse types of technology were included in our measurement.

Although the link between older age and being a nonowner or nonuser of mHealth technologies is consistently reported across studies, we recommend continued population-level research of mHealth nonowners and nonusers because the proportion of digital natives will grow as the population ages. In turn, this may increase the proportion of the population that trusts collecting personal health information on digital devices [26]. Perceived utility of mHealth technologies for health or behavioral tracking may also increase among nonowners and nonusers and former owners and users as mHealth technologies advance in their functionality, especially if approaches such as ensuring sociocultural relevancy [27] and person-centered design [28,29] are considered throughout mHealth technology development.

In addition, our findings related to mHealth nonowners and nonusers suggest that clinical and public health practitioners could consider alternatives to mHealth interventions to track and promote health behavior change, especially among less educated older men. However, they also call to mind the importance of ensuring that not owning or using mHealth technologies for health or behavioral tracking is not due to barriers that can be addressed, such as digital health literacy [28,30-32]. Researchers and organizations are addressing some of these issues through programs such as RecycleHealth [31,33], American Association of Retired Persons Tek workshops [34,35], and The Wellness Group [36], with measurable beneficial effects on health and behavioral outcomes [31,36].

#### Characteristics of Mobile Health Owners and Users by Pattern of Mobile Health Use for Health and Behavioral Tracking

In this study, we also discovered differences in the characteristics of mHealth owners and users by comparing nontrackers, app trackers, and device trackers with the largest group of mHealth owners and users, supertrackers. Supertrackers, those who use multiple devices for health or behavioral tracking and goal setting, make up approximately one-quarter of the US population. Our findings demonstrate that supertrackers, conceivably the most intrinsically interested in mHealth technologies, are younger than nontrackers and device trackers, who have in common that they do not use mHealth technologies to track a health-related goal. Supertrackers also tended to be female when compared with their tracker counterparts, device trackers and app trackers. This finding related to gender is consistent with previous studies of people who download smartphone health apps [24] and users of wearable activity monitors, such as Fitbits [5].

With respect to socioeconomic factors, when compared with supertrackers, we found that being an app tracker or being a device tracker, respectively, was associated with reporting an annual household income in the US $20,000-US $49,999 range vs US $75,000 or more. Paré et al [7] found in a Canadian sample that reporting the highest annual income level, greater than Can $80,000 (US $59968.80), was associated with being a *digital self-tracker*, someone who uses health apps, wearables, or digital medical devices. By analyzing the US population’s distinctive patterns of mHealth use, we add to the literature that multiuse of mHealth technologies for health or behavioral tracking and goal setting is associated with higher reported annual household income. In addition, level of education was not significantly associated with mHealth owners and users’ pattern of mHealth use for health and behavioral tracking when controlling all other factors. Other studies have found that higher level of education is associated with having or downloading smartphone health apps [2,24,37], digital self-tracking [7], and reporting current use of a wearable activity monitor [5]. Future studies should continue to examine relationships between mHealth owners and users’ specific pattern of mHealth use, education, and income, as ease of use, pricing, and accessibility will continue to evolve with the advancement of mHealth technologies.

Although we did not find that racial or ethnic identity was associated with being an mHealth owner or user vs being in the mHealth nonowners and nonusers category (see Aim 2 results), race and ethnicity was associated with mHealth owners and users’ pattern of mHealth use for health and behavioral tracking. For example, non-Hispanic blacks and respondents in the other race and ethnicity category had lower odds of being device trackers than supertrackers. Future studies should explore the relationship between sociocultural factors that foster or challenge interest and motivation to use mHealth technologies and the perceived sociocultural relevancy of different types of mHealth technologies for health or behavioral tracking and goal setting [27].

We also discovered several health-related characteristics associated with mHealth owners and users’ pattern of mHealth use for health and behavioral tracking. For example, we found that supertrackers tended to be health information seekers when compared with mHealth owners and users who do not use devices to track health goals (ie, nontrackers and device trackers). One explanation for this finding is that supertrackers may be relatively more attentive, interested, and curious about personal health information (evidenced by their goal-setting behavior), including collecting and recording it on digital devices [30,38]. Health information–seeking behavior and goal setting appear to be distinguishing characteristics among the US population’s mHealth technology users.

Unlike the results of our analysis of mHealth nonowners and nonusers vs mHealth owners and users, we found a significant relationship between mHealth owners and users’ pattern of mHealth use for health and behavioral tracking and weight status. Specifically, supertrackers had greater odds of being overweight or obese compared with nontrackers and device trackers, respectively. Krebs and Duncan [2] found that smartphone owners who download health apps are more likely to be overweight or obese, and Byuhan et al [37] also report greater odds of using health apps to track a health-related goal among obese compared with underweight individuals. Therefore, we add to the findings of these authors that being overweight or obese is associated with multiuse of mHealth technologies for health or behavioral tracking and goal setting.

Having a chronic condition (diabetes, hypertension, or a heart condition) was also associated with mHealth owners and users’ pattern of mHealth use for health and behavioral tracking; however, the direction of the relationship differed when comparing supertrackers with app trackers and with device trackers. Namely, supertrackers had greater odds of having a chronic condition than app trackers but lower odds of having a chronic condition than device trackers. This result may be explained by the wide range of devices other than a smartphone or tablet that respondents might use, ranging from fitness trackers to digital medical devices, such as glucometers. On the basis of the characteristics of device trackers—male, older age, non–health information seekers, with a chronic condition—the type of mHealth technologies used by this group may be digital medical devices over fitness trackers. We recommend that future iterations of HINTS include separate items to measure use of specific types of wearables and other portable digital devices because motivations for use and individual differences of mHealth technology users likely vary considerably by the specific type of nonsmartphone or nontablet mHealth technology used.

We also recommend that mHealth intervention researchers and health care providers who study or prescribe mHealth to individuals who fit the device tracker profile consider individual-level factors that might threaten continued engagement with mHealth technologies, such as relatively less intrinsic interest in digital self-tracking, potentially low digital health literacy, and data-entry burden [2,29,36]. However, our findings suggest opportunities, not only potential challenges. For example, although device trackers in our study reported that they do not use health apps to track health-related goals, about one-third reported downloading health apps onto their smartphone or tablet. These downloads may represent implementation intentions that clinical and public health practitioners might leverage by working with device trackers to set personal health goals within their health apps. Health goal-setting and reminders embedded in mHealth technologies that stimulate habit formation may lead to *guided mastery*, which is theorized to help people with implementation intentions act on their behavioral intentions [22].

Notably, geographical area (urban vs rural), perceived health, and health self-efficacy were not significantly associated with pattern of mHealth use for health and behavioral tracking. These results are inconsistent with studies showing that mHealth technology users are more likely to reside in urban over rural areas [37]. One explanation for our findings may be the rapid increase in smartphone ownership throughout the entire United States since the time other population studies were conducted [1]. Although better perceived health status [7,10,24,29,37] and greater health self-efficacy [37] have been associated with mHealth technology use in past studies, our results highlight that other individual-level factors may be stronger predictors when mHealth technology owners and users are compared by distinctive pattern of mHealth use for health and behavioral tracking.

### Limitations

This study is the largest and most current nationally representative study of mHealth ownership and use in US adults across 2 cycles of NCI HINTS. However, there are also several limitations, including reliance on self-report and cross-sectional data. Because the survey data are cross-sectional, we cannot infer causal relationships between variables (eg, being a supertracker leads to obesity or vice versa). In addition, we cannot discern whether respondents intended to engage, reengage, or disengage from mHealth technologies following their participation in HINTS.

Given the limitations of the dataset, we could neither determine the type of devices used beyond smartphones and tablets nor discern whether respondents used a combination of nonsmartphone or nontablet devices (eg, fitness tracker plus a digital medical device). We also could not distinguish their specific reasons for tracking health or behaviors (eg, blood glucose monitoring or number of steps walked) or the extent to which they engage with mHealth technologies (frequency of use, duration of use, etc). Moreover, we were unable to evaluate their experience of mHealth technology use (eg, usability and acceptability). However, findings from this study may be used to guide development of future research focused on improving users’ experience of mHealth technologies for health or behavioral tracking because we were able to capture the characteristics of the US population by diverse patterns of mHealth use.

An additional limitation of this study is that we could not determine the specific health goals respondents set in their health apps. Goals may have varied from smoking cessation to weight loss to asthma control, for example. Although the aim of this study was not to make predictions based on the specific goals of mHealth technology use, we acknowledge that making such distinctions is important for behavioral interventions.

### Conclusions

This study contributes to understanding of the US adult population’s ownership, use (including multiuse), and nonuse of diverse types of mHealth technologies for health or behavioral tracking and goal setting. We discovered that characteristics, such as age, gender, being a health information seeker, and having a chronic condition, are associated with specific patterns of mHealth use. Researchers and clinical and public health practitioners can apply these findings to research design, practice, and health message development to better reach intended audiences and promote health behavior change.

Although mHealth technologies have the potential to broadly reach people and facilitate behavior change, our findings suggest that an appreciation for the diverse ways mHealth technologies are used (and not used) for health or behavioral tracking and goal setting should be considered when designing as well as interpreting the results of mHealth intervention studies. Future studies could build on this work through continued surveillance of patterns of mHealth use for health and behavioral tracking and the individual-level factors associated with those patterns. Research that keeps pace with mHealth technology development is needed to understand the contextual factors that help explain variation in population-level effects of mHealth technology use on health behavior.

## Appendix

Multimedia Appendix 1 Health Information National Trends Survey 5, Cycle 1 instrument.

Multimedia Appendix 2 Health Information National Trends Survey 5, Cycle 2 instrument.

Multimedia Appendix 3 Weighted Population Estimates for Sociodemographic and Health-Related Characteristics, HINTS 5, Cycle 1 and Cycle 2.

## Abbreviations

HINTS: Health Information National Trends Survey
mHealth: mobile health
NCI: National Cancer Institute
OR: odds ratio

## References

[1] Taylor K, Silver L (2019). Pew Research Center.

[2] Krebs P, Duncan DT (2015). Health app use among US mobile phone owners: a national survey. JMIR Mhealth Uhealth.

[3] Dunn J, Runge R, Snyder M (2018). Wearables and the medical revolution. Per Med.

[4] Hitlin P (2018). Pew Research Center.

[5] Omura JD, Carlson SA, Paul P, Watson KB, Fulton JE (2017). National physical activity surveillance: users of wearable activity monitors as a potential data source. Prev Med Rep.

[6] Jiang S, Hong YA, Liu PL (2019). Trends of online patient-provider communication among cancer survivors from 2008 to 2017: a digital divide perspective. J Cancer Surviv.

[7] Paré G, Leaver C, Bourget C (2018). Diffusion of the digital health self-tracking movement in Canada: results of a national survey. J Med Internet Res.

[8] Yingling LR, Mitchell V, Ayers CR, Peters-Lawrence M, Wallen GR, Brooks AT, Troendle JF, Adu-Brimpong J, Thomas S, Henry J, Saygbe JN, Sampson DM, Johnson AA, Graham AP, Graham LA, Wiley KL, Powell-Wiley T (2017). Adherence with physical activity monitoring wearable devices in a community-based population: observations from the Washington, D.C., Cardiovascular Health and Needs Assessment. Transl Behav Med.

[9] Tuman M, Moyer A (2019). Health intentions and behaviors of health app owners: a cross-sectional study. Psychol Health Med.

[10] Robbins R, Krebs P, Jagannathan R, Jean-Louis G, Duncan DT (2017). Health app use among US mobile phone users: analysis of trends by chronic disease status. JMIR Mhealth Uhealth.

[11] Wang Y, Xue H, Huang Y, Huang L, Zhang D (2017). A systematic review of application and effectiveness of mhealth interventions for obesity and diabetes treatment and self-management. Adv Nutr.

[12] Brickwood KJ, Watson G, O'Brien J, Williams AD (2019). Consumer-based wearable activity trackers increase physical activity participation: systematic review and meta-analysis. JMIR Mhealth Uhealth.

[13] Schoeppe S, Alley S, van Lippevelde W, Bray NA, Williams SL, Duncan MJ, Vandelanotte C (2016). Efficacy of interventions that use apps to improve diet, physical activity and sedentary behaviour: a systematic review. Int J Behav Nutr Phys Act.

[14] Qudah B, Luetsch K (2019). The influence of mobile health applications on patient - healthcare provider relationships: a systematic, narrative review. Patient Educ Couns.

[15] Buman MP, Epstein DR, Gutierrez M, Herb C, Hollingshead K, Huberty JL, Hekler EB, Vega-López S, Ohri-Vachaspati P, Hekler AC, Baldwin CM (2016). BeWell24: development and process evaluation of a smartphone 'app' to improve sleep, sedentary, and active behaviors in US Veterans with increased metabolic risk. Transl Behav Med.

[16] Carpenter DM, Geryk LL, Sage A, Arrindell C, Sleath BL (2016). Exploring the theoretical pathways through which asthma app features can promote adolescent self-management. Transl Behav Med.

[17] Desveaux L, Shaw J, Saragosa M, Soobiah C, Marani H, Hensel J, Agarwal P, Onabajo N, Bhatia RS, Jeffs L (2018). A mobile app to improve self-management of individuals with type 2 diabetes: qualitative realist evaluation. J Med Internet Res.

[18] Gordon JS, Armin J, Hingle MD, Giacobbi P, Cunningham JK, Johnson T, Abbate K, Howe CL, Roe DJ (2017). Development and evaluation of the See Me Smoke-Free multi-behavioral mHealth app for women smokers. Transl Behav Med.

[19] Hesse BW, Beckjord E, Ahern DK, Hilliard ME, Riekert KA, Ockene JK, Pbert L (2018). Role of technology in behavior change to expand reach and impact on public health. The Handbook of Health Behavior Change. Fifth Edition.

[20] Locke EA, Latham GP (2002). Building a practically useful theory of goal setting and task motivation. A 35-year odyssey. Am Psychol.

[21] Perski O, Blandford A, West R, Michie S (2017). Conceptualising engagement with digital behaviour change interventions: a systematic review using principles from critical interpretive synthesis. Transl Behav Med.

[22] Pirolli P, Youngblood GM, Du H, Konrad A, Nelson L, Springer A (2018). Scaffolding the mastery of healthy behaviors with Fittle+ systems: Evidence-based interventions and theory. Hum–Comput Interact.

[23] Samdal GB, Eide GE, Barth T, Williams G, Meland E (2017). Effective behaviour change techniques for physical activity and healthy eating in overweight and obese adults; systematic review and meta-regression analyses. Int J Behav Nutr Phys Act.

[24] Carroll JK, Moorhead A, Bond R, LeBlanc WG, Petrella RJ, Fiscella K (2017). Who uses mobile phone health apps and does use matter? A secondary data analytics approach. J Med Internet Res.

[25] Rutten LJ, Davis T, Beckjord EB, Blake K, Moser RP, Hesse BW (2012). Picking up the pace: changes in method and frame for the health information national trends survey (2011-2014). J Health Commun.

[26] Serrano KJ, Yu M, Riley WT, Patel V, Hughes P, Marchesini K, Atienza AA (2016). Willingness to exchange health information via mobile devices: Findings from a population-based survey. Ann Fam Med.

[27] Abroms LC, Allegrante JP, Auld ME, Gold RS, Riley WT, Smyser J (2019). Toward a common agenda for the public and private sectors to advance digital health communication. Am J Public Health.

[28] Kreps GL (2017). The relevance of health literacy to mHealth. Inform Serv Use.

[29] Simblett S, Greer B, Matcham F, Curtis H, Polhemus A, Ferrão J, Gamble P, Wykes T (2018). Barriers to and facilitators of engagement with remote measurement technology for managing health: systematic review and content analysis of findings. J Med Internet Res.

[30] Cho J, Park D, Lee HE (2014). Cognitive factors of using health apps: systematic analysis of relationships among health consciousness, health information orientation, eHealth literacy, and health app use efficacy. J Med Internet Res.

[31] Gualtieri L, Phillips J, Rosenbluth S, Synoracki S (2018). Digital literacy: A barrier to adoption of connected health technologies in older adults. iproc.

[32] Tennant B, Stellefson M, Dodd V, Chaney B, Chaney D, Paige S, Alber J (2015). eHealth literacy and web 2.0 health information seeking behaviors among baby boomers and older adults. J Med Internet Res.

[33] RecycleHealth.

[34] American Association of Retired Persons.

[35] Milani RV, Bober RM, Lavie CJ (2016). The role of technology in chronic disease care. Prog Cardiovasc Dis.

[36] Gualtieri L, Rosenbluth S, Phillips J (2016). Can a free wearable activity tracker change behavior? The impact of trackers on adults in a physician-led wellness group. JMIR Res Protoc.

[37] Bhuyan SS, Lu N, Chandak A, Kim H, Wyant D, Bhatt J, Kedia S, Chang CF (2016). Use of mobile health applications for health-seeking behavior among US adults. J Med Syst.

[38] O'Brien HL, Toms EG (2008). What is user engagement? A conceptual framework for defining user engagement with technology. J Am Soc Inf Sci Technol.

