# Supplementary material for: Characterizing the US Population by Patterns of Mobile Health Use for Health and Behavioral Tracking: Analysis of the National Cancer Institute's Health Information National Trends Survey Data
Source: J Med Internet Res. 2020 May 14;22(5):e16299. doi: 10.2196/16299 (PMC7256752; doi:10.2196/16299)
Supplement: Multimedia Appendix 1 [file jmir_v22i5e16299_app1.pdf]

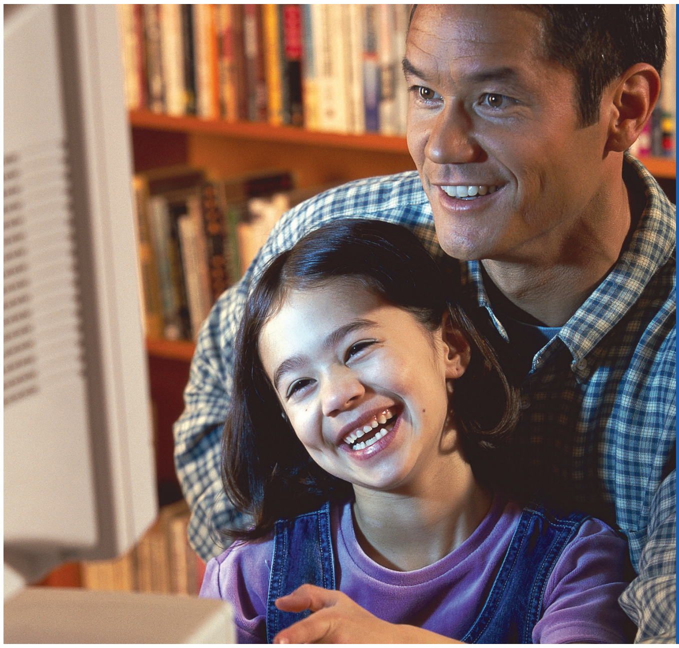

Health Information

National Trends Survey

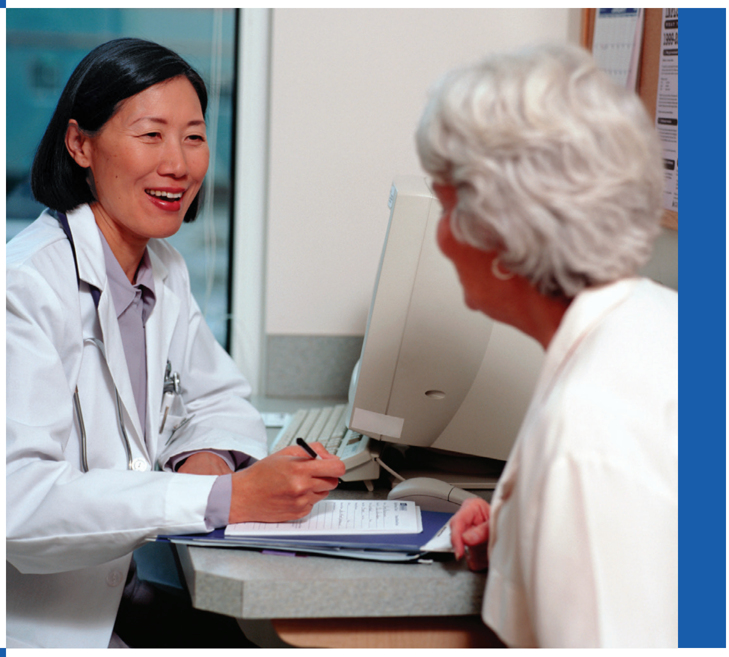

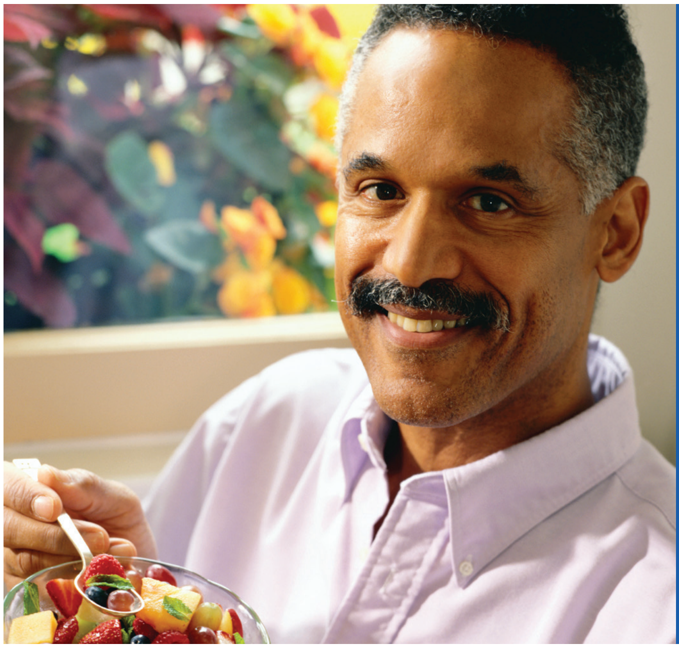

National Institutes of Health
U.S. Department of Health and Human Services

DEPARTMENT OF HEALTH & HUMAN SERVICES • USA

40769.0614.88610402

HV-C1

Annotated Form
Cycle1, Next-Birthday, Full-Content, English Version

START HERE:

1. Is there more than one person age 18 or older living in this household?

AdultsInHH

- 1 Yes
- 2 No → **GO TO A1 on the next page**

2. Including yourself, how many people age 18 or older live in this household?

MailHHAdults

☐☐

3. **The adult with the next birthday should complete this questionnaire.** This way, across all households, HINTS will include responses from adults of all ages.

4. Please write the first name, nickname, or initials of the adult with the next birthday. This is the person who should complete the questionnaire.

☐

Si prefiere recibir la encuesta en español, por favor llame 1-888-738-6812

A: Looking For Health Information

A1. Have you ever looked for information about health or medical topics from any source?

SeekHealthInfo

- 1 Yes
- 2 No → **GO TO A5 in the next column**

A2. The most recent time you looked for information about health or medical topics, where did you go first? WhereSeekHealthInfo

Mark ☒ only one.

- 1 Books
- 2 Brochures, pamphlets, etc.
- 3 Cancer organization
- 4 Family
- 5 Friend/Co-worker
- 6 Doctor or health care provider
- 7 Internet
- 8 Library
- 9 Magazines
- 10 Newspapers
- 11 Telephone information number
- 12 Complementary, alternative, or unconventional practitioner

WhereSeekHealthInfo_IMP

A3. The most recent time you looked for information about health or medical topics, who was it for?

WhoLookingFor

- 1 Myself
- 2 Someone else
- 3 Both myself and someone else

A4. Based on the results of your most recent search for information about health or medical topics, how much do you agree or disagree with each of the following statements?

	Strongly agree	Somewhat agree	Somewhat disagree	Strongly disagree
a. It took a lot of effort to get the information you needed............ LotOfEffort	1	2	3	4
b. You felt frustrated during your search for the information......... Frustrated	1	2	3	4
c. You were concerned about the quality of the information........... ConcernedQuality	1	2	3	4
d. The information you found was hard to understand.................. TooHardUnderstand	1	2	3	4

A5. Overall, how confident are you that you could get advice or information about health or medical topics if you needed it?

ConfidentGetHealthInf

- 1 Completely confident
- 2 Very confident
- 3 Somewhat confident
- 4 A little confident
- 5 Not confident at all

A6. In general, how much would you trust information about health or medical topics from each of the following?

	Not at all	A little	Some	A lot
a. A doctor................................. TrustDoctor	4	3	2	1
b. Family or friends..................... TrustFamily	4	3	2	1
c. Newspapers or magazines...... TrustNewsMag	4	3	2	1
d. Radio.. TrustRadio	4	3	2	1
e. Internet..................................... TrustInternet	4	3	2	1
f. Television.................................. TrustTelevison	4	3	2	1
g. Government health agencies... TrustGov	4	3	2	1
h. Charitable organizations.......... TrustCharities	4	3	2	1
i. Religious organizations and leaders.................................. TrustReligiousOrgs	4	3	2	1

5168

A7. Imagine that you had a strong need to get information about health or medical topics. Where would you go first?

Mark ☒ only one. StrongNeedHealthInfo

- 1 Books
- 2 Brochures, pamphlets, etc.
- 3 Cancer organization
- 4 Family
- 5 Friend/Co-worker
- 6 Doctor or health care provider
- 7 Internet
- 8 Library
- 9 Magazines
- 10 Newspapers
- 11 Telephone information number
- 12 Complementary, alternative, or unconventional practitioner
- 91 Other-Specify → StrongNeedHealthInfo_OS

StrongNeedHealthInfo_IMP

A8. Have you ever looked for information about cancer from any source?

SeekCancerInfo

- 1 Yes
- 2 No

A9. In the past 12 months, have you used the Internet to look for information about cancer for yourself?

InternetCancerInfoSelf

- 1 Yes
- 2 No

B: Using the Internet to Find Information

B1. Do you ever go on-line to access the Internet or World Wide Web, or to send and receive e-mail?

UseInternet

- 1 Yes
- 2 No → **GO TO B4 on the next page**

B2. When you use the Internet, do you access it through...

	Yes	No
a. A regular dial-up telephone line.................... Internet_DialUp	1	2
b. Broadband such as DSL, cable, or FiOS..... Internet_BroadBnd	1	2
c. A cellular network (i.e., phone, 3G/4G)........ Internet_Cell	1	2
d. A wireless network (Wi-Fi)........................... Internet_Wifi	1	2

B3. How often do you access the Internet through each of the following?

	Daily	Sometimes	Never	Not applicable
a. Computer at home...................... WhereUseInternet_Home	1	2	3	4
b. Computer at work....................... WhereUseInternet_Work	1	2	3	4
c. Computer at school.................... WhereUseInternet_School	1	2	3	4
d. Computer in a public place (library, community center, other)... WhereUseInternet_PublicPlace	1	2	3	4
e. On a mobile device (cell phone/smart phone/tablet)......... WhereUseInternet_MobileDevice	1	2	3	4
f. On a gaming device/ "Smart TV".. WhereUseInternet_GamingDevice	1	2	3	4

B4. In the past 12 months, have you used a computer, smartphone, or other electronic means to do any of the following…

	Yes	No
a. Looked for health or medical information for yourself......... Electronic_SelfHealthInfo	1	2
b. Looked for health or medical information for someone else......... Electronic_HealthInfoSE	1	2
c. Bought medicine or vitamins online......... Electronic_BuyMedicine	1	2
d. Looked for a health care provider......... Electronic_HCPSearch	1	2
e. Used e-mail or the Internet to communicate with a doctor or a doctor's office......... Electronic_TalkDoctor	1	2
f. Made appointments with a health care provider......... Electronic_MadeAppts	1	2
g. Track health care charges and costs......... Electronic_TrackedHealthCosts	1	2
h. Filled out forms or paperwork related to your health care......... Electronic_CompletedForms	1	2
i. Look up test results......... Electronic_TestResults	1	2

B5. Please indicate if you have each of the following.

	Yes	No
a. Tablet computer like an iPad, Samsung Galaxy, Motorola Xoom, or Kindle Fire......... HaveDevice_Tablet	1	2
b. Smartphone, such as an iPhone, Android, Blackberry, or Windows phone......... HaveDevice_SmartPh	1	2
c. Basic cell phone only......... HaveDevice_CellPh	1	2

B6. On your tablet or smartphone, do you have any "apps" related to health and wellness?

TabletHealthWellnessApps

1 Yes

2 No

3 Don't know

4 Do not have a tablet or smartphone → **GO TO B8 in the next column**

B7. Has your tablet or smartphone…

	Yes	No
a. Helped you track progress on a health-related goal such as quitting smoking, losing weight, or increasing physical activity?......... Tablet_AchieveGoal	1	2
b. Helped you make a decision about how to treat an illness or condition?......... Tablet_MakeDecision	1	2
c. Helped you in discussions with your health care provider?......... Tablet_DiscussionsHCP	1	2

B8. Other than a tablet or smartphone, have you used an electronic device to monitor or track your health within the last 12 months? Examples include Fitbit, blood glucose meters, and blood pressure monitors.

OtherDevTrackHealth

1 Yes

2 No

B9. Have you shared health information from either an electronic monitoring device or smartphone with a health professional within the last 12 months?

SharedHealthDeviceInfo

1 Yes

2 No

3 Not Applicable

B10. Sometimes people use the Internet to connect with other people online through social networks like Facebook or Twitter. This is often called "social media".

In the last 12 months, have you used the Internet for any of the following reasons?

	Yes	No
a. To visit a social networking site, such as Facebook or LinkedIn......... IntRSN_VisitedSocNet	1	2
b. To share health information on social networking sites, such as Facebook or Twitter......... IntRSN_SharedSocNet	1	2
c. To write in an online diary or blog (i.e., Web log)......... IntRSN_WroteBlog	1	2
d. To participate in an online forum or support group for people with a similar health or medical issue......... IntRSN_SupportGroup	1	2
e. To watch a health-related video on YouTube......... IntRSN_YouTube	1	2

5168

B11. Have you sent or received a text message from a doctor or other health care professional within the last 12 months? TextFromDoctor

1 Yes
2 No
3 Don't know

C: Your Health Care

C1. Not including psychiatrists and other mental health professionals, is there a particular doctor, nurse, or other health professional that you see most often? RegularProvider

1 Yes
2 No

C2. About how long has it been since you last visited a doctor for a routine checkup? A routine checkup is a general physical exam, not an exam for a specific injury, illness, or condition. MostRecentCheckup2

1 Within the past year
2 1-2 years ago
3 3-5 years ago
4 More than 5 years ago
5 Never
6 Don't know

C3. In the past 12 months, not counting times you went to an emergency room, how many times did you go to a doctor, nurse, or other health professional to get care for yourself? FreqGoProvider

0 None → **GO TO C7 on the next page**
1 1 time
2 2 times
3 3 times
4 4 times
5 5-9 times
6 10 or more times

C4. The following questions are about your communication with all doctors, nurses, or other health professionals you saw during the past 12 months.

How often did they do each of the following:	Always	Usually	Sometimes	Never
a. Give you the chance to ask all the health-related questions you had........ ChanceAskQuestions	1	2	3	4
b. Give the attention you needed to your feelings and emotions........... FeelingsAddresed	1	2	3	4
c. Involve you in decisions about your health care as much as you wanted........ InvolvedDecisions	1	2	3	4
d. Make sure you understood the things you needed to do to take care of your health........ UnderstoodNextSteps	1	2	3	4
e. Explain things in a way you could understand........ ExplainedClearly	1	2	3	4
f. Spend enough time with you........ SpentEnoughTime	1	2	3	4
g. Help you deal with feelings of uncertainty about your health or health care........ HelpUncertainty	1	2	3	4

C5. Overall, how would you rate the quality of health care you received in the past 12 months? QualityCare

1 Excellent
2 Very good
3 Good
4 Fair
5 Poor

5168

C6. In the past 12 months, when getting care for a medical problem, was there a time when you...

	Yes	No
a. Had to bring an X-ray, MRI, or other type of test result with you to the appointment? ProbCare_BringTest	1	2
b. Had to wait for test results longer than you thought reasonable? ProbCare_WaitLong	1	2
c. Had to redo a test or procedure because the earlier test results were not available? ProbCare_RedoTest	1	2
d. Had to provide your medical history again because your chart could not be found? ProbCare_ProvideHist	1	2

C7. Are you currently covered by any of the following types of health insurance or health coverage plans?

	Yes	No
a. Insurance through a current or former employer or union HealthIns_InsuranceEMP	1	2
b. Insurance purchased directly from an insurance company HealthIns_InsurancePriv	1	2
c. Medicare, for people 65 and older, or people with certain disabilities HealthIns_Medicare	1	2
d. Medicaid, Medical Assistance, or any kind of government-assistance plan for those with low incomes or a disability HealthIns_Medicaid	1	2
e. TRICARE or other military health care HealthIns_Tricare	1	2
f. VA (including those who have ever used or enrolled for VA health care) HealthIns_VA	1	2
g. Indian Health Service HealthIns_IHS	1	2
h. Any other type of health insurance or health coverage plan (specify) HealthIns_Other	1	2

→ HealthIns_Other_OS

D: Medical Records

Next, we are going to ask you some questions about your medical records. Medical records are defined as medical history, such as laboratory test results, clinical notes, and current list of medications.

D1. Do any of your doctors or other health care providers maintain your medical records in a computerized system? ProviderMaintainEMR2

1 Yes
2 No
3 Don't know

D2. Have you ever been offered online access to your medical records by your…

	Yes	No	Don't know
a. health care provider? OfferedAccessHCP2	1	2	3
b. health insurer? OfferedAccessInsurer2	1	2	3

D3. Have any of your health care providers, including doctors, nurses, or office staff ever encouraged you to use an online medical record? HCPEncourageOnlineRec

1 Yes
2 No

D4. How many times did you access your online medical record in the last 12 months? AcccessOnlineRecord

- [0] 0
- [1] 1 to 2 times
- [2] 3 to 5 times
- [3] 6 to 9 times
- [4] 10 or more times

(1–4: **GO TO D6 in the next column**)

D5. Why have you not accessed your medical records online? Is it because…

	Yes	No
a. You prefer to speak to your health care provider directly?............ NotAccessed_SpeakDirectly	1	2
b. You do not have a way to access the website?............ NotAccessed_NoInternet	1	2
c. You did not have a need to use your online medical record?............ NotAccessed_NoNeed	1	2
d. You were concerned about the privacy or security of the website that had your medical records?............ NotAccessed_ConcernedPrivacy	1	2
e. You don't have an online medical record.... NotAccessed_NoRecord	1	2
f. Other (specify)............	1	2

NotAccessed_Other
NotAccessed_Other_OS

If you have not accessed any medical records in the last 12 months, go to D12 on the next page.

Otherwise, go to D6 in the next column

D6. Do any of your online medical records include the following types of medical information?

	Yes	No	Don't know
a. Laboratory test results............ RecordsOnline_Labs	1	2	3
b. Current list of medications............ RecordsOnline_Meds	1	2	3
c. List of health/medical problems............ RecordsOnline_HealthProbs	1	2	3
d. Allergy list............ RecordsOnline_Allergies	1	2	3
e. Summaries of your office visit............ RecordsOnline_VisitSummary	1	2	3
f. Clinical notes............ RecordsOnline_ClinNotes	1	2	3
g. Immunization or vaccination history.. RecordsOnline_Immunizations	1	2	3

D7. In the past 12 months, have you used your online medical record to…

	Yes	No
a. Make appointments with a health care provider?............ RecordsOnline_MakeAppt	1	2
b. Request refill of medications?............ RecordsOnline_RefillMeds	1	2
c. Fill out forms or paperwork related to your health care?............ RecordsOnline_Paperwork	1	2
d. Request correction of inaccurate information?............ RecordsOnline_RequestCorrection	1	2

D8. In the past 12 months, have you used your online medical record to…

	Yes	No
a. Securely message health care provider and staff (e.g., e-mail)............ RecordsOnline_MessageHCP	1	2
b. Look up test results............ RecordsOnline_ViewResults	1	2
c. Monitor your health............ RecordsOnline_MonitorHealth	1	2
d. Download your health information to your computer or mobile device, such as a cell phone or tablet............ RecordsOnline_DownloadHealth	1	2
e. Add health information to share with your health care provider, such as health concerns, symptoms, and side-effects............ RecordsOnline_AddHealthInfo	1	2
f. Help you make a decision about how to treat an illness or condition............ RecordsOnline_MakeDecision	1	2

5168

D9. Have you electronically sent your medical information to….?

	Yes	No
a. Another health care provider?...................... ESent_AnotherHCP	1	2
b. A family member or another person involved with your care?.............................. ESent_Family	1	2
c. A service or app that can help manage and store your health information?............... ESent_HealthApp	1	2

D10. How easy or difficult was it to understand the health information in your online medical record? UnderstandOnlineMedRec

1 Very easy
2 Somewhat easy
3 Somewhat difficult
4 Very difficult

D11. In general, how useful is your online medical record for monitoring your health? UsefulOnlineMedRec

1 Very useful
2 Somewhat useful
3 Not very useful
4 Not at all useful
5 I do not use my online medical record to monitor my health

D12. How confident are you that safeguards (including the use of technology) are in place to protect your medical record from being seen by people who aren't permitted to see them? ConfidentInfoSafe

1 Very confident
2 Somewhat confident
3 Not confident

D13. Have you ever kept information from your health care provider because you were concerned about the <u>privacy</u> or <u>security</u> of your medical record? WithheldInfoPrivacy

1 Yes
2 No

D14. If your medical information is sent <u>electronically</u> – that is, by computer – from one health care provider to another, how concerned are you that an unauthorized person would see it? ElectInfoSafe

1 Very concerned
2 Somewhat concerned
3 Not concerned

D15. How many times did you access a <u>family member or close friend's</u> online medical record in the last 12 months? AccessFamilyMedRec

0 None ➔ **GO TO E1 below**
1 1 to 2 times
2 3 to 5 times
3 6 to 9 times
4 10 or more times

D16. How did you access a family member or close friend's personal health information?

	Yes	No
a. Used family member's login and password.. AccessedFamRec_TheirPwd	1	2
b. Used a login and password assigned to me to access their record............................ AccessedFamRec_MyPwd	1	2

E: Caregiving

E1. Are you currently caring for or making health care decisions for someone with a medical, behavioral, disability, or other condition?

Mark ☒ <u>all that apply</u>.

1 Yes, a child/children Caregiving_Child
1 Yes, a spouse/partner Caregiving_Spouse
1 Yes, a parent/parents Caregiving_Parent
1 Yes, a close family member, Caregiving_Family
1 Yes, a friend or other non-relative Caregiving_Friend
1 No ➔ **GO TO F1 on the next page** Caregiving_No
CaregivingWho_Cat

E2. Please check all conditions for which you have provided care for this person.

If you selected more than one person in E1, please think about the individual for whom you have provided the most care.

Mark ☒ all that apply.

- 1 **Cancer** Caregiving_Cancer
- 1 **Alzheimer's, confusion, dementia, forgetfulness** Caregiving_Alzheimers
- 1 **Orthopedic/Musculoskeletal Issues** (examples: back problems, broken bones, arthritis, mobility problems, can't get around, feeble, unsteady, falling) Caregiving_OrthoMusc
- 1 **Mental Health/Behavioral/Substance Abuse Issues** (examples: mental illness, emotional problems, depression, anxiety, substance/drug/alcohol abuse) Caregiving_MentalHealth
- 1 **Chronic Conditions** (examples: high blood pressure/hypertension, diabetes, heart disease, heart attack, lung disease, emphysema, Chronic Obstructive Pulmonary Disease (COPD), Parkinson's) Caregiving_ChronicCond
- 1 **Neurological/Developmental Issues** (examples: brain damage or injury, developmental or intellectual disorder, mental retardation, Down syndrome, stroke) Caregiving_NeuroDev
- 1 **Acute Conditions** (examples: surgery, wounds/injuries) Caregiving_AcuteCond
- 1 **Aging/Aging related health issues** Caregiving_Aging
- 1 **Other (specify)** → Caregiving_Other Caregiving_Other_OS
- 1 **Not sure/Don't know** Caregiving_NotSure

CaregivingCond_Cat

E3. Thinking of all of the kinds of help you provide for this person or persons, about how many hours do you spend in an average week providing care? Caregiving_HoursPerWeek

- 1 Less than 5 hours per week
- 2 5-14 hours per week
- 3 15-20 hours per week
- 4 21-34 hours per week
- 5 35 or more hours per week

F: Medical Research

F1. Doctors use DNA tests to analyze someone's DNA for health reasons. Have you heard or read about this type of genetic test? HeardDNATest

- 1 Yes
- 2 No → **GO TO G1 on the next page**

F2. Which of the following uses of a genetic test have you heard of?

Mark ☒ all that apply.

- 1 Determining risk or likelihood of getting a particular disease GeneticTestUse_DetermineRisk
- 1 Determining how a disease should be treated after diagnosis GeneticTestUse_DetermineTx
- 1 Determining which drug(s) may or may not work for an individual GeneticTestUse_DetermineMed
- 1 Determining the likelihood of passing an inherited disease to your children GeneticTestUse_DeterminePass

GeneticTestUse_Cat

F3. Have you ever had any of the following type(s) of genetic tests?

Mark ☒ all that apply.

- 1 **Paternity testing:** To determine if a man is the father of a child HadTest_Paternity
- 1 **Ancestry testing:** To determine the background or geographic/ethnic origin of an individual's ancestors HadTest_Ancestry
- 1 **DNA fingerprinting:** To distinguish between or match individuals using hair, blood, or other biological material HadTest_DNAFing
- 1 **Cystic Fibrosis (CF) carrier testing:** To determine if a person is at risk of having a child with cystic fibrosis HadTest_CFCarrier
- 1 **BRCA 1/2 testing:** To determine if a person has more than an average chance of developing breast cancer or ovarian cancer HadTest_BRCA
- 1 **Lynch syndrome testing:** To determine if a person has more than an average chance of developing colon cancer HadTest_Lynch
- 1 None of the above HadTest_None
- 1 Not sure HadTest_NotSure
- 1 Other-Specify → HadTest_Other, HadTest_Other_OS

HadTest_Cat

5168

G: Your Overall Health

G1. In general, would you say your health is...
GeneralHealth

- 1 Excellent,
- 2 Very good,
- 3 Good,
- 4 Fair, or
- 5 Poor?

G2. Overall, how confident are you about your ability to take good care of your health?
OwnAbilityTakeCareHealth

- 1 Completely confident
- 2 Very confident
- 3 Somewhat confident
- 4 A little confident
- 5 Not confident at all

G3. Has a doctor or other health professional ever told you that you had any of the following medical conditions:

	Yes	No
a. Diabetes or high blood sugar?...................... MedConditions_Diabetes	1	2
b. High blood pressure or hypertension?.......... MedConditions_HighBP	1	2
c. A heart condition such as heart attack, angina, or congestive heart failure?............. MedConditions_HeartCondition	1	2
d. Chronic lung disease, asthma, emphysema, or chronic bronchitis?.............. MedConditions_LungDisease	1	2
e. Arthritis or rheumatism?................................ MedConditions_Arthritis	1	2
f. Depression or anxiety disorder?.................. MedConditions_Depression	1	2

G4. About how tall are you without shoes?

☐ Feet ***and*** ☐☐ Inches
Height_Feet, Height_Inches

G5. About how much do you weigh, in pounds, without shoes? Weight

☐☐☐ Pounds

G6. <u>Over the past 2 weeks</u>, how often have you been bothered by any of the following problems?

	Nearly every day	More than half the days	Several days	Not at all
a. Little interest or pleasure in doing things............................ LittleInterest	1	2	3	4
b. Feeling down, depressed, or hopeless................................ Hopeless	1	2	3	4
c. Feeling nervous, anxious, or on edge................................. Nervous	1	2	3	4
d. Not being able to stop or control worrying..................... Worrying	1	2	3	4

G7. Is there anyone you can count on to provide you with emotional support when you need it - such as talking over problems or helping you make difficult decisions?
EmotionalSupport

- 1 Yes
- 2 No

G8. Do you have friends or family members that you talk to about your health?
TalkHealthFriends

- 1 Yes
- 2 No

G9. If you needed help with your daily chores, is there someone who can help you?
HelpDailyChores

- 1 Yes
- 2 No

G10. Are you deaf or do you have serious difficulty hearing?
Deaf

- 1 Yes
- 2 No

5168

H: Health and Nutrition

H1. When available, how often do you use menu information on calories in deciding what to order? UseMenuCalorieInfo

- 1 Always
- 2 Often
- 3 Sometimes
- 4 Rarely
- 5 Never

H2. About how many cups of fruit (including 100% pure fruit juice) do you eat or drink each day? Fruit

- 0 None
- 1 ½ cup or less
- 2 ½ cup to 1 cup
- 3 1 to 2 cups
- 4 2 to 3 cups
- 5 3 to 4 cups
- 6 4 or more cups

1 cup of fruit could be:
- 1 small apple
- 1 large banana
- 1 large orange
- 8 large strawberries
- 1 medium pear
- 2 large plums
- 32 seedless grapes
- 1 cup (8 oz.) fruit juice
- ½ cup dried fruit
- 1 inch-thick wedge of watermelon

H3. About how many cups of vegetables (including 100% pure vegetable juice) do you eat or drink each day? Vegetables

- 0 None
- 1 ½ cup or less
- 2 ½ cup to 1 cup
- 3 1 to 2 cups
- 4 2 to 3 cups
- 5 3 to 4 cups
- 6 4 or more cups

1 cup of vegetables could be:
- 3 broccoli spears
- 1 cup cooked leafy greens
- 2 cups lettuce or raw greens
- 12 baby carrots
- 1 medium potato
- 1 large sweet potato
- 1 large ear of corn
- 1 large raw tomato
- 2 large celery sticks
- 1 cup of cooked beans

H4. Which of the following health conditions do you think can result from drinking too much alcohol?

	Yes	No	Don't know
a. Cancer........ AlcoholConditions_Cancer	1	2	3
b. Heart Disease........ AlcoholConditions_HeartDisease	1	2	3
c. Diabetes........ AlcoholConditions_Diabetes	1	2	3
d. High cholesterol........ AlcoholConditions_Cholesterol	1	2	3
e. Liver disease........ AlcoholConditions_LiverDisease	1	2	3
f. Being overweight or obese........ AlcoholConditions_Overweight	1	2	3

H5. How much do you agree or disagree with each of the following statements?

	Strongly agree	Somewhat agree	Somewhat disagree	Strongly disagree
a. Alcohol increases your risk of cancer........ AlcoholIncreaseCancer	1	2	3	4
b. Drinking alcohol in moderation reduces your risk of heart disease........ AlcoholReduceHeart	1	2	3	4

5168

I: Physical Activity, Exercise, and UV Exposure

I1. In a typical week, how many days do you do any physical activity or exercise of at least moderate intensity, such as brisk walking, bicycling at a regular pace, and swimming at a regular pace?

- 0 None → **GO TO I4 in the next column**
- 1 1 day per week TimesModerateExercise
- 2 2 days per week
- 3 3 days per week
- 4 4 days per week
- 5 5 days per week
- 6 6 days per week
- 7 7 days per week

I2. On the days that you do any physical activity or exercise of at least moderate intensity, how long do you typically do these activities?

Write a number in one box below.

[ ][ ] Minutes [ ][ ] Hours

HowLongModerateExerciseMn
HowLongModerateExerciseHr

I3. In a typical week, outside of your job or work around the house, how many days do you do leisure-time physical activities specifically designed to strengthen your muscles such as lifting weights or circuit training (do not include cardio exercise such as walking, biking, or swimming)?

TimesStrengthTraining

- 0 None
- 1 1 day per week
- 2 2 days per week
- 3 3 days per week
- 4 4 days per week
- 5 5 days per week
- 6 6 days per week
- 7 7 days per week

I4. How many times in the past 12 months have you used a tanning bed or booth?

TanningBed

- 0 0 times
- 1 1 to 2 times
- 2 3 to 10 times
- 3 11 to 24 times
- 4 25 or more times

I5. Do you ever have your skin examined by a health professional for signs of skin cancer? SkinCancerHPExam

- 2 No
- 1 Yes, but not regularly
- 3 Yes, regularly
- 4 I don't know

I6. Do you ever check your skin for signs of skin cancer? SkinCancerSelfCheck

- 2 No
- 1 Yes, but not regularly
- 3 Yes, regularly

J: Tobacco Products

J1. Have you smoked at least 100 cigarettes in your entire life? Smoke100

- 1 Yes
- 2 No → **GO TO J5 on the next page**

J2. How often do you now smoke cigarettes?

SmokeNow

- 1 Every day
- 2 Some days
- 3 Not at all → **GO TO J5 on the next page**

J3. At any time in the past year, have you stopped smoking for one day or longer because you were trying to quit?

TriedQuit

- 1 Yes
- 2 No

5168

J4. Are you seriously considering quitting smoking in the next six months?

ConsiderQuit

1 Yes
2 No

J5. New types of cigarettes are now available called electronic cigarettes (also known as e-cigarettes or personal vaporizers). These products deliver nicotine through a vapor. Compared to smoking cigarettes, would you say that electronic cigarettes are …

ElectCigLessHarm

1 Much less harmful,
2 Less harmful,
3 Just as harmful,
4 More harmful,
5 Much more harmful, or
6 I've never heard of electronic cigarettes. → **GO TO J8 below**

J6. Have you ever used an e-cigarette, even one or two times?

UsedECigEver

1 Yes
2 No → **GO TO J8 below**

J7. Do you now use an e-cigarette every day, some days, or not at all?

UseECigNow

1 Every day
2 Some days
3 Not at all

J8. At any time in the past year, have you talked with your doctor or other health professional about having a test to check for lung cancer?

DrTalkLungTest

1 Yes
2 No
3 Don't know

J9. In your opinion, do you think that some smokeless tobacco products, such as chewing tobacco, snus, and snuff are less harmful to a person's health than cigarettes?

SmokelessLessHarm

1 Yes
2 No
3 Don't know

J10. A hookah pipe (or shisha) is a large water pipe. People smoke tobacco using hookah pipes in groups at cafes or bars. Compared to smoking cigarettes, would you say that smoking tobacco using a hookah is…

HookahLessHarm

1 Much less harmful,
2 Less harmful,
3 Just as harmful,
4 More harmful,
5 Much more harmful, or
6 I've never heard of Hookah.

K: Screening for Cancer

K1. Are you male or female?

GenderC

1 Male → **GO TO K4 below**
2 Female

K2. How long ago did you have your most recent Pap test to check for cervical cancer?

WhenPapTest

1 A year ago or less
2 More than 1, up to 2 years ago
3 More than 2, up to 3 years ago
4 More than 3, up to 5 years ago
5 More than 5 years ago
6 I have never had a Pap test

K3. When did you have your most recent mammogram to check for breast cancer, if ever? WhenMammogram

1 A year ago or less
2 More than 1, up to 2 years ago
3 More than 2, up to 3 years ago
4 More than 3, up to 5 years ago
5 More than 5 years ago
6 I have never had a mammogram

Females GO TO L1 on the next page

K4. A PSA test is used to check for prostate cancer.

Have you ever had a PSA test?

EverHadPSATest

1 Yes
2 No

5168

L: HPV Awareness

L1. Have you ever heard of HPV? HPV stands for Human Papillomavirus. It is not HIV, HSV, or herpes.

HeardHPV

1 Yes
2 No → **GO TO L5 below**

L2. Do you think HPV can cause…

	Yes	No	Not sure
a. Cervical Cancer?............................... HPVCauseCancer_Cervical	1	2	3
b. Penile Cancer?................................. HPVCauseCancer_Penile	1	2	3
c. Anal Cancer?.................................... HPVCauseCancer_Anal	1	2	3
d. Oral Cancer?.................................... HPVCauseCancer_Oral	1	2	3

L3. Do you think that HPV is a sexually transmitted disease (STD)?

HPVSTD

1 Yes
2 No
3 Not sure

L4. Do you think HPV requires medical treatment or will it usually go away on its own without treatment?

HPVMedicalTreatment

1 Requires medical treatment
2 Will usually go away on its own

L5. A vaccine to prevent HPV infection is available and is called the HPV shot, cervical cancer vaccine, GARDASIL®, or Cervarix®.

Before today, have you ever heard of the cervical cancer vaccine or HPV shot?

HeardHPVVaccine2

1 Yes
2 No

L6. In your opinion, how successful is the HPV vaccine at preventing cervical cancer?

HPVShotPrevent

1 Not at all successful
2 A little successful
3 Pretty successful
4 Very successful
5 Don't know

L7. Including yourself, is anyone in your immediate family between the ages of 9 and 27 years old?

FamBetween9and27

1 Yes
2 No → **GO TO M1 on the next page**

L8. In the last 12 months, has a doctor or health care professional ever talked with you or an immediate family member about the HPV shot or vaccine?

DiscussHPVVaccination12m

1 Yes
2 No
3 Don't know

L9. In the last 12 months, has a doctor or health care professional recommended that you or someone in your immediate family get an HPV shot or vaccine?

RecommendHPVShot

1 Yes
2 No
3 Don't know

5168

M: Your Cancer History

M1. Have you ever been diagnosed as having cancer? EverHadCancer

1 Yes
2 No → **GO TO N1 on the next page**

M2. What type of cancer did you have?

Mark ☒ all that apply.

1 Bladder cancer CaBladder
1 Bone cancer CaBone
1 Breast cancer CaBreast
1 Cervical cancer (cancer of the cervix) CaCervical
1 Colon cancer CaColon
1 Endometrial cancer (cancer of the uterus) CaEndometrial
1 Head and neck cancer CaHeadNeck
1 Hodgkin's lymphoma CaHodgkins
1 Leukemia/Blood cancer CaLeukemia
1 Liver cancer CaLiver
1 Lung cancer CaLung
1 Melanoma CaMelanoma
1 Non-Hodgkin lymphoma CaNonHodgkin
1 Oral cancer CaOral
1 Ovarian cancer CaOvarian
1 Pancreatic cancer CaPancreatic
1 Pharyngeal (throat) cancer CaPharyngeal
1 Prostate cancer CaProstate
1 Rectal cancer CaRectal
1 Renal (kidney) cancer CaRenal
1 Skin cancer, non-melanoma CaSkin
1 Stomach cancer CaStomach
1 Other-Specify → CaOther, CaOther_OS

Cancer_Cat

M3. At what age were you first told that you had cancer? WhenDiagnosedCancer

[ ][ ][ ] Age

M4. Did you ever receive any treatment for your cancer? UndergoCancerTreatment

1 Yes
2 No → **GO TO M8 in the next column**

M5. Which of the following cancer treatments have you ever received?

	**Yes**	**No**
a. Chemotherapy (IV or pills) CancerTx_Chemo	1	2
b. Radiation CancerTx_Radiation	1	2
c. Surgery CancerTx_Surgery	1	2
d. Other CancerTx_Other	1	2

M6. About how long ago did you receive your last cancer treatment? HowLongFinishTreatment_Cat

5 Still receiving treatment → **GO TO M10 on the next page**
1 Less than 1 year ago
2 1 year ago to less than 5 years ago
3 5 years ago to less than 10 years ago
4 10 or more years ago

M7. Did you ever receive a summary document from your doctor or other health care professional that listed all of the treatments you received for your cancer? CancerTxSummary

1 Yes
2 No

M8. Were you ever denied health insurance coverage because of your cancer? CancerDeniedCoverage

1 Yes
2 No

M9. Looking back, since the time you were first diagnosed with cancer, how much, if at all, has cancer and its treatment hurt your financial situation? CancerHurtFinances

1 Not at all
2 A little
3 Some
4 A lot

5168

M10. At any time since you were first diagnosed with cancer, did any doctor or other health care provider ever discuss with you the impact of cancer or its treatment on your ability to work? CancerAbilityToWork

1 Discussed it with me in detail
2 Briefly discussed it with me
3 Did not discuss it at all
4 I don't remember
5 I was not working at the time of my diagnosis

M11. Clinical trials are research studies that involve people. They are designed to test the safety and effectiveness of new treatments and to compare new treatments with the standard care that people currently get. Have you ever participated in a clinical trial for treatment of your cancer? ClinicalTrialCancerTx

1 Yes
2 No
3 Don't know

M12. Has a doctor or other member of your medical team discussed clinical trials as a treatment option for your cancer? DiscussedClinicalTrial

1 Yes
2 No

N: Beliefs About Cancer

▶ Think about cancer in general when answering the questions in this section.

N1. How likely are you to get cancer in your lifetime? ChanceGetCancer

1 Very unlikely
2 Unlikely
3 Neither unlikely nor likely
4 Likely
5 Very likely

N2. How much do you agree or disagree with each of the following statements?

	Strongly agree	Somewhat agree	Somewhat disagree	Strongly disagree
a. It seems like everything causes cancer....................................... EverythingCauseCancer	1	2	3	4
b. There's not much you can do to lower your chances of getting cancer....................................... PreventNotPossible	1	2	3	4
c. There are so many different recommendations about preventing cancer, it's hard to know which ones to follow........... TooManyRecommendations	1	2	3	4
d. In adults, cancer is more common than heart disease........ CancerMoreCommon	1	2	3	4
e. When I think about cancer, I automatically think about death....................................... CancerFatal	1	2	3	4

N3. How much do you agree or disagree with this statement: "I'd rather not know my chance of getting cancer." RatherNotKnowChance

1 Strongly agree
2 Somewhat agree
3 Somewhat disagree
4 Strongly disagree

N4. How worried are you about getting cancer? FreqWorryCancer

1 Not at all
2 Slightly
3 Somewhat
4 Moderately
5 Extremely

5168

N5. Have any of your family members ever had cancer? FamilyEverHadCancer

1 Yes
2 No
4 Not sure

O: You and Your Household

O1. What is your age? Age

[| |] Years old

O2. What is your current occupational status?

Mark ☒ only one. OccupationStatus

1 Employed Employed
2 Unemployed Unemployed
3 Homemaker Homemaker
4 Student Student
5 Retired Retired
6 Disabled Disabled
91 Other-Specify → OccupationStatus_OS

OtherOcc
MultiOcc

O3. Have you ever served on active duty in the U.S. Armed Forces, military Reserves or National Guard? Active duty does not include training in the Reserves or National Guard, but DOES include activation, for example, for the Persian Gulf War. ActiveDutyArmedForces

1 Yes, now on active duty
2 Yes, on active duty in the last 12 months but not now
3 Yes, on active duty in the past, but not in the last 12 months
4 No, training for Reserves or National Guard only
5 No, never served in the military

GO TO O5 in the next column

O4. In the past 12 months, have you received some or all of your health care from a VA hospital or clinic? ReceivedCareVA

1 Yes, all of my health care
2 Yes, some of my health care
3 No, no VA health care received

O5. What is your marital status?

Mark ☒ only one. MaritalStatus

1 Married
2 Living as married
3 Divorced
4 Widowed
5 Separated
6 Single, never been married

O6. What is the highest grade or level of schooling you completed? Education

1 Less than 8 years
2 8 through 11 years
3 12 years or completed high school
4 Post high school training other than college (vocational or technical)
5 Some college
6 College graduate
7 Postgraduate

O7. Were you born in the United States? BornInUSA

1 Yes → **GO TO O9 below**
2 No

O8. In what year did you come to live in the United States? YearCameToUSA

[| | |] Year

O9. How well do you speak English? SpeakEnglish

1 Very well
2 Well
3 Not well
4 Not at all

5168

O10. Are you of Hispanic, Latino/a, or Spanish origin? One or more categories may be selected.

Mark ☒ all that apply.

- 1 No, not of Hispanic, Latino/a, or Spanish origin NotHisp
- 1 Yes, Mexican, Mexican American, Chicano/a Mexican
- 1 Yes, Puerto Rican PuertoRican
- 1 Yes, Cuban Cuban
- 1 Yes, another Hispanic, Latino/a, or Spanish origin OthHisp

Hisp_Cat

O11. What is your race? One or more categories may be selected.

Mark ☒ all that apply.

- 1 White White
- 1 Black or African American Black
- 1 American Indian or Alaska Native AmerInd
- 1 Asian Indian AsInd
- 1 Chinese Chinese
- 1 Filipino Filipino
- 1 Japanese Japanese
- 1 Korean Korean
- 1 Vietnamese Vietnamese
- 1 Other Asian OthAsian
- 1 Native Hawaiian Hawaiian
- 1 Guamanian or Chamorro Guamanian
- 1 Samoan Samoan
- 1 Other Pacific Islander OthPacIsl

Race_Cat2

O12. Do you think of yourself as… SexualOrientation

- 1 Heterosexual, or straight
- 2 Homosexual, or gay or lesbian
- 3 Bisexual
- 91 Something else – Specify → SexualOrientation_OS

SexualOrientation_I

O13. Including yourself, how many people live in your household? TotalHousehold

☐☐ Number of people

O14. Starting with yourself, please mark the sex, and write in the age and month of birth for each adult 18 years of age or older living at this address.

	Sex	Age	Month Born (01-12)
SELF (SelfGender)	1 Male / 2 Female	SelfAge	SelfMOB
Adult 2 (HHAdultGender2)	1 Male / 2 Female	HHAdultAge2	HHAdultMOB2
Adult 3 (HHAdultGender3)	1 Male / 2 Female	HHAdultAge3	HHAdultMOB3
Adult 4 (HHAdultGender4)	1 Male / 2 Female	HHAdultAge4	HHAdultMOB4
Adult 5 (HHAdultGender5)	1 Male / 2 Female	HHAdultAge5	HHAdultMOB5

O15. How many children under the age of 18 live in your household? ChildrenInHH

☐☐ Number of children under 18

O16. Do you currently rent or own your home? RentOrOwn

- 1 Own
- 2 Rent
- 3 Occupied without paying monetary rent

O17. Does anyone in your family have a working cell phone? CellPhone

- 1 Yes
- 2 No

O18. Is there at least one telephone inside your home that is currently working and is not a cell phone? PhoneInHome

- 1 Yes
- 2 No

5168

O19. Thinking about members of your family living in this household, what is your combined annual income, meaning the total pre-tax income from all sources earned in the past year?

IncomeRanges

- 1 $0 to $9,999
- 2 $10,000 to $14,999
- 3 $15,000 to $19,999
- 4 $20,000 to $34,999
- 5 $35,000 to $49,999
- 6 $50,000 to $74,999
- 7 $75,000 to $99,999
- 8 $100,000 to $199,999
- 9 $200,000 or more

O20. About how long did it take you to complete the survey?

Write a number in <u>one</u> box below.

☐☐ Minutes ☐☐ Hours

MailSurveyTimeMin
MailSurveyTimeHrs

O21. At which of the following types of addresses does your household currently receive residential mail?

Mark ☒ <u>all that apply</u>.

- 1 A street address with a house or building number TypeOfAddressA
- 1 An address with a rural route number TypeOfAddressB
- 1 A U.S. post office box (P.O. Box) TypeOfAddressC
- 1 A commercial mailbox establishment (such as Mailboxes R Us, and Mail Boxes Etc.®) TypeOfAddressD

Thank you!

▶ Please return this questionnaire in the postage-paid envelope within 2 weeks.

▶ If you have lost the envelope, mail the completed questionnaire to:

HINTS Study, TC 1046F
Westat
1600 Research Boulevard
Rockville, MD 20850

5168
