# Supplementary material for: Characterizing the US Population by Patterns of Mobile Health Use for Health and Behavioral Tracking: Analysis of the National Cancer Institute's Health Information National Trends Survey Data
Source: J Med Internet Res. 2020 May 14;22(5):e16299. doi: 10.2196/16299 (PMC7256752; doi:10.2196/16299)
Supplement: Multimedia Appendix 2 [file jmir_v22i5e16299_app2.pdf]

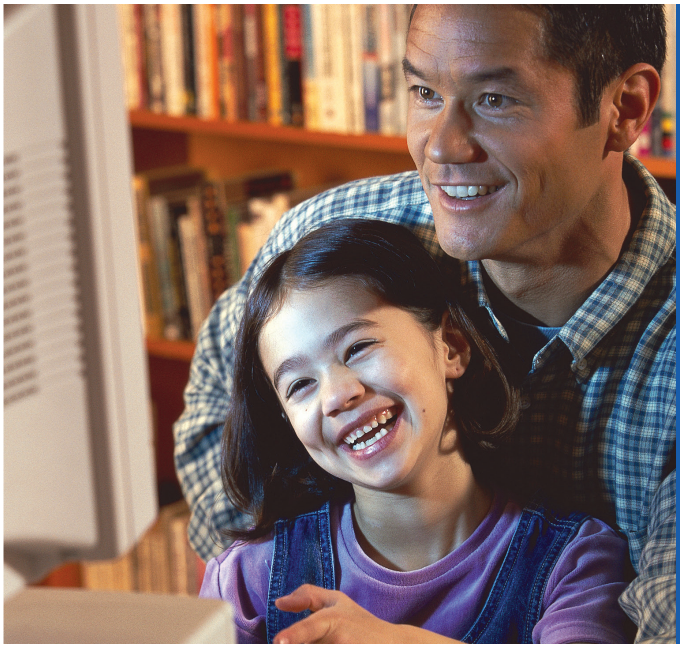

Health Information

National Trends Survey

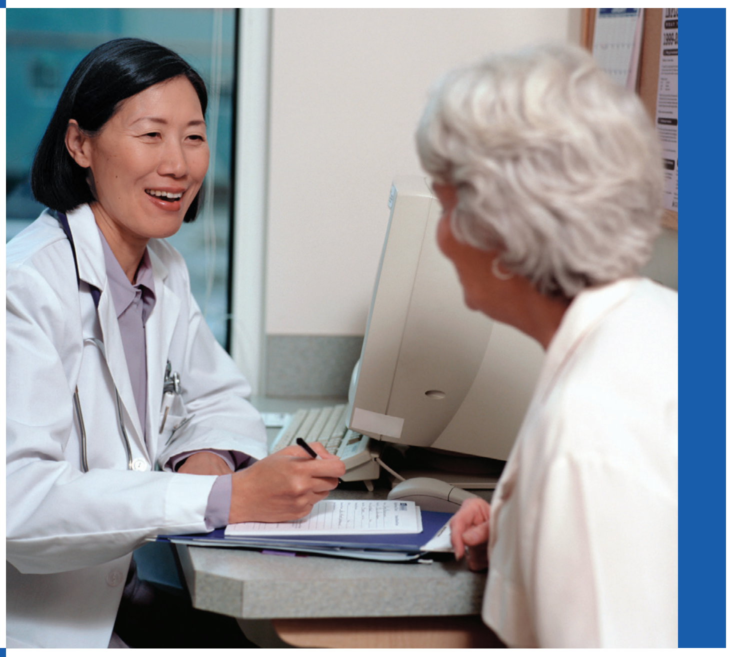

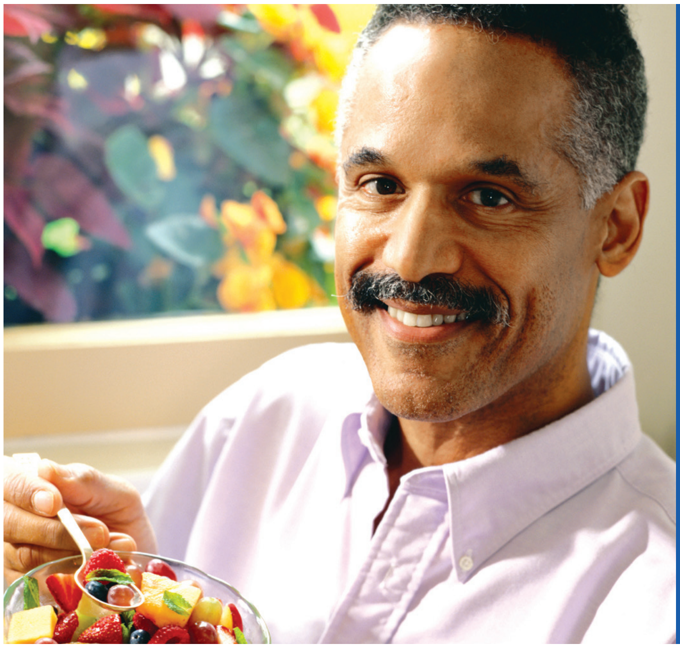

National Institutes of Health
U.S. Department of Health and Human Services

H5–C2

Annotated Form
Cycle 2, Full-Content, English Version

START HERE:

Instructions

- Please use a black or blue pen to complete this form.
- Mark ☒ to indicate your answer.
- If you want to change your answer, mark ■ on the wrong answer.

1. Is there more than one person age 18 or older living in this household?

AdultsInHH

☐ 1 Yes
☐ 2 No → **GO TO A1 on the next page**

2. Including yourself, how many people age 18 or older live in this household?

MailHHAdults

☐☐

3. **The adult with the next birthday should complete this questionnaire.** This way, across all households, HINTS will include responses from adults of all ages.

4. Please write the first name, nickname, or initials of the adult with the next birthday. This is the person who should complete the questionnaire.

☐

Si prefiere recibir la encuesta en español, por favor llame 1-888-738-6812

55346

A: Looking For Health Information

A1. Have you ever looked for information about health or medical topics from any source? SeekHealthInfo

- 1 Yes
- 2 No → **GO TO A6 in the next column**

A2. The most recent time you looked for information about health or medical topics, where did you go first? WhereSeekHealthInfo

Mark only one.

- 1 Books
- 2 Brochures, pamphlets, etc.
- 3 Cancer organization
- 4 Family
- 5 Friend/Co-worker
- 6 Doctor or health care provider
- 7 Internet
- 8 Library
- 9 Magazines
- 10 Newspapers
- 11 Telephone information number
- 12 Complementary, alternative, or unconventional practitioner

WhereSeekHealthInfo_IMP

A3. The most recent time you looked for information about health or medical topics, who was it for? WhoLookingFor

- 1 Myself
- 2 Someone else
- 3 Both myself and someone else

A4. Have you ever looked for information about cancer from any source? SeekCancerInfo

- 1 Yes
- 2 No → **GO TO A6 below**

A5. Based on the results of your most recent search for information about cancer, how much do you agree or disagree with each of the following statements?

	Strongly agree	Somewhat agree	Somewhat disagree	Strongly disagree
a. It took a lot of effort to get the information you needed............ CancerLotOfEffort	1	2	3	4
b. You felt frustrated during your search for the information......... CancerFrustrated	1	2	3	4
c. You were concerned about the quality of the information........... CancerConcernedQuality	1	2	3	4
d. The information you found was hard to understand.................... CancerTooHardUnderstand	1	2	3	4

A6. Overall, how confident are you that you could get advice or information about cancer if you needed it? CancerConfidentGetHealthInf

- 1 Completely confident
- 2 Very confident
- 3 Somewhat confident
- 4 A little confident
- 5 Not confident at all

55346

A7. In general, how much would you trust information about cancer from each of the following?

	Not at all	A little	Some	A lot
a. A doctor................................ CancerTrustDoctor	4	3	2	1
b. Family or friends..................... CancerTrustFamily	4	3	2	1
c. Newspapers or magazines...... CancerTrustNewsMag	4	3	2	1
d. Radio....................................... CancerTrustRadio	4	3	2	1
e. Internet.................................... CancerTrustInternet	4	3	2	1
f. Television................................ CancerTrustTelevison	4	3	2	1
g. Government health agencies... CancerTrustGov	4	3	2	1
h. Charitable organizations.......... CancerTrustCharities	4	3	2	1
i. Religious organizations and leaders.................................. CancerTrustReligiousOrgs	4	3	2	1

A8. Imagine that you had a strong need to get information about cancer. Where would you go first? StrongNeedCancerInfo

Mark only one.

1 Books
2 Brochures, pamphlets, etc.
3 Cancer organization
4 Family
5 Friend/Co-worker
6 Doctor or health care provider
7 Internet
8 Library
9 Magazines
10 Newspapers
11 Telephone information number
12 Complementary, alternative, or unconventional practitioner
91 Other – Specify → StrongNeedCancerInfo_OS

StrongNeedCancerInfo_IMP

B: Using the Internet to Find Information

B1. Do you ever go on-line to access the Internet or World Wide Web, or to send and receive e-mail? UseInternet

1 Yes
2 No → **GO TO B5 on the next page**

B2. When you use the Internet, do you access it through...

	Yes	No
a. A regular dial-up telephone line.................... Internet_DialUp	1	2
b. Broadband such as DSL, cable, or FiOS..... Internet_BroadBnd	1	2
c. A cellular network (i.e., phone, 3G/4G)........ Internet_Cell	1	2
d. A wireless network (Wi-Fi).......................... Internet_WiFi	1	2

B3. In the past 12 months, have you used the Internet to look for information about cancer for yourself? InternetCancerInfoSelf

1 Yes
2 No

B4. How often do you access the Internet through each of the following?

	Daily	Sometimes	Never	Not Applicable
a. Computer at home...................... WhereUseInternet_Home	1	2	3	4
b. Computer at work....................... WhereUseInternet_Work	1	2	3	4
c. Computer in a public place (library, community center, other).. WhereUseInternet_PublicPlace	1	2	3	4
d. On a mobile device (cell phone/smart phone/tablet)......... WhereUseInternet_MobileDevice	1	2	3	4

55346

B5. In the past 12 months, have you used a computer, smartphone, or other electronic means to do any of the following?

	Yes	No
a. Looked for health or medical information for yourself............................. Electronic_SelfHealthInfo	1	2
b. Looked for health or medical information for someone else.................... Electronic_HealthInfoSE	1	2
c. Bought medicine or vitamins online.......... Electronic_BuyMedicine	1	2
d. Looked for assistance for the care that you provide for someone else........... Electronic_LookedAssistance	1	2
e. Used e-mail or the Internet to communicate with a doctor or a doctor's office.. Electronic_TalkDoctor	1	2
f. Tracked health care charges and costs..... Electronic_TrackedHealthCosts	1	2
g. Looked up medical test results.................. Electronic_TestResults	1	2

B6. Please indicate if you have each of the following.

Mark all that apply.

- 1 Tablet computer (for example, an iPad, Samsung Galaxy, Motorola Xoom, or Kindle Fire) HaveDevice_Tablet
- 1 Smartphone (for example, an iPhone, Android, Blackberry, or Windows phone) HaveDevice_SmartPh
- 1 Basic cell phone only HaveDevice_CellPh
- 1 I do not have any of the above HaveDevice_None

GO TO B9 in the next column

HaveDevice_Cat

B7. On your tablet or smartphone, do you have any "apps" related to health and wellness? TabletHealthWellnessApps

- 1 Yes
- 2 No
- 3 Don't know

B8. Has your tablet or smartphone…

	Yes	No
a. Helped you track progress on a health-related goal such as quitting smoking, losing weight, or increasing physical activity?...................................... Tablet_AchieveGoal	1	2
b. Helped you make a decision about how to treat an illness or condition?................. Tablet_MakeDecision	1	2
c. Helped you in discussions with your health care provider?................................ Tablet_DiscussionsHCP	1	2

B9. Other than a tablet or smartphone, have you used an electronic device to monitor or track your health within the last 12 months? Examples include Fitbit, blood glucose meters, and blood pressure monitors. OtherDevTrackHealth

- 1 Yes
- 2 No

B10. Have you shared health information from either an electronic monitoring device or smartphone with a health professional within the last 12 months? SharedHealthDeviceInfo

- 1 Yes
- 2 No
- 3 Not Applicable

55346

B11. Sometimes people use the Internet to connect with other people online through social networks like Facebook or Twitter. This is often called "social media".

In the past 12 months, have you used the Internet for any of the following reasons?

	Yes	No
a. To visit a social networking site, such as Facebook or LinkedIn.................................. IntRsn_VisitedSocNet	1	2
b. To share health information on social networking sites, such as Facebook or Twitter.. IntRsn_SharedSocNet	1	2
c. To write in an online diary or blog (i.e., Web log).. IntRsn_WroteBlog	1	2
d. To participate in an online forum or support group for people with a similar health or medical issue................................ IntRsn_SupportGroup	1	2
e. To watch a health-related video on YouTube... IntRsn_YouTube	1	2

B12. Have you sent a text message to or received a text message from a doctor or other health care professional within the last 12 months? TextFromDoctor

1 Yes
2 No
3 Don't know

C: Your Health Care

C1. Not including psychiatrists and other mental health professionals, is there a particular doctor, nurse, or other health professional that you see most often? RegularProvider

1 Yes
2 No

C2. About how long has it been since you last visited a doctor for a routine checkup? A routine checkup is a general physical exam, not an exam for a specific injury, illness, or condition. MostRecentCheckup2

1 Within the past year
2 1-2 years ago
3 3-5 years ago
4 More than 5 years ago
5 Never
6 Don't know

C3. In the past 12 months, not counting times you went to an emergency room, how many times did you go to a doctor, nurse, or other health professional to get care for yourself? FreqGoProvider

0 None → **GO TO C7 on the next page**
1 1 time
2 2 times
3 3 times
4 4 times
5 5-9 times
6 10 or more times

C4. The following questions are about your communication with all doctors, nurses, or other health professionals you saw during the past 12 months.

How often did they do each of the following?

	Always	Usually	Sometimes	Never
a. Give you the chance to ask all the health-related questions you had ChanceAskQuestions	1	2	3	4
b. Give the attention you needed to your feelings and emotions FeelingsAddressed	1	2	3	4
c. Involve you in decisions about your health care as much as you wanted InvolvedDecisions	1	2	3	4
d. Make sure you understood the things you needed to do to take care of your health UnderstoodNextSteps	1	2	3	4
e. Explain things in a way you could understand ExplainedClearly	1	2	3	4
f. Spend enough time with you SpentEnoughTime	1	2	3	4
g. Help you deal with feelings of uncertainty about your health or health care HelpUncertainty	1	2	3	4

C5. Overall, how would you rate the quality of health care you received in the past 12 months? QualityCare

1 Excellent
2 Very good
3 Good
4 Fair
5 Poor

C6. In the last 12 months, when getting care for a medical problem, was there a time when you...

	Yes	No
a. Had to bring an X-ray, MRI, or other type of test result with you to the appointment? ProbCare_BringTest	1	2
b. Had to wait for test results longer than you thought reasonable? ProbCare_WaitLong	1	2
c. Had to redo a test or procedure because the earlier test results were not available? ProbCare_RedoTest	1	2
d. Had to provide your medical history again because your chart could not be found? ProbCare_ProvideHist	1	2

C7. Are you currently covered by any of the following types of health insurance or health coverage plans?

	Yes	No
a. Insurance through a current or former employer or union HealthIns_InsuranceEmp	1	2
b. Insurance purchased directly from an insurance company HealthIns_InsurancePriv	1	2
c. Medicare, for people 65 and older, or people with certain disabilities HealthIns_Medicare	1	2
d. Medicaid, Medical Assistance, or any kind of government-assistance plan for those with low incomes or a disability HealthIns_Medicaid	1	2
e. TRICARE or other military health care HealthIns_Tricare	1	2
f. VA (including those who have ever used or enrolled for VA health care) HealthIns_VA	1	2
g. Indian Health Service HealthIns_IHS	1	2
h. Any other type of health insurance or health coverage plan (Specify) HealthIns_Other	1	2

HealthIns_Other_OS

55346

D: Medical Records

Next, we are going to ask you some questions about your medical records. Medical records are defined as medical history, such as laboratory test results, clinical notes, and current list of medications.

D1. Do any of your doctors or other health care providers maintain your medical records in a computerized system? ProviderMaintainEMR2

- 1 Yes
- 2 No
- 3 Don't Know

D2. How confident are you that safeguards (including the use of technology) are in place to protect your medical records from being seen by people who aren't permitted to see them? ConfidentInfoSafe

- 1 Very confident
- 2 Somewhat confident
- 3 Not confident

D3. Have you ever kept information from your health care provider because you were concerned about the privacy or security of your medical record? WithheldInfoPrivacy

- 1 Yes
- 2 No

D4. Have you ever been offered online access to your medical records by your health care provider or health insurer? EverOfferedAccessRec

- 1 Yes
- 2 No → **GO TO E1 on the next page**
- 3 Don't Know → **GO TO E1 on the next page**

D5. Who offered you online access to your medical records?

Mark all that apply.

- 1 Health care provider WhoOffered_HCP
- 1 Health insurer WhoOffered_Insurer
- 1 Something else – Specify → WhoOffered_Other WhoOffered_Other_OS

WhoOffered_Cat

D6. How many times did you access your online medical record in the last 12 months? AccessOnlineRecord

- 0 0 → **GO TO D7 below**
- 1 1 to 2 times
- 2 3 to 5 times
- 3 6 to 9 times
- 4 10 or more times

(1–4) **GO TO D8 on the next page**

D7. Why have you not accessed your medical record online? Is it because…

	Yes	No
a. You prefer to speak to your health care provider directly? NotAccessed_SpeakDirectly	1	2
b. You do not have a way to access the website? NotAccessed_NoInternet	1	2
c. You did not have a need to use your online medical record? NotAccessed_NoNeed	1	2
d. You were concerned about the privacy or security of the website that had your medical records? NotAccessed_ConcernedPrivacy	1	2
e. You don't have an online medical record? NotAccessed_NoRecord	1	2
f. Other (Specify) → NotAccessed_Other NotAccessed_Other_OS	1	2

If you have not accessed any medical records in the last 12 months, go to E1 on the next page.

Otherwise, go to D8

55346

D8. In the past 12 months, have you used your online medical record to...

	Yes	No
a. Request refill of medications? RecordsOnline_RefillMeds	1	2
b. Fill out forms or paperwork related to your health care? RecordsOnline_Paperwork	1	2
c. Request correction of inaccurate information? RecordsOnline_RequestCorrection	1	2
d. Securely message health care provider and staff (for example, e-mail)? RecordsOnline_MessageHCP	1	2
e. Download your health information to your computer or mobile device, such as a cell phone or tablet? RecordsOnline_DownloadHealth	1	2
f. Add health information to share with your health care provider, such as health concerns, symptoms, and side effects? RecordsOnline_AddHealthInfo	1	2
g. Help you make a decision about how to treat an illness or condition? RecordsOnline_MakeDecision	1	2

D9. Do any of your online medical records include the following types of medical information?

	Yes	No	Don't know
a. List of health/medical problems RecordsOnline_HealthProbs	1	2	3
b. Allergy list RecordsOnline_Allergies	1	2	3
c. Summaries of your office visit RecordsOnline_VisitSummary	1	2	3
d. Clinical notes RecordsOnline_ClinNotes	1	2	3
e. Immunization or vaccination history RecordsOnline_Immunizations	1	2	3

D10. Have you electronically sent your medical information to….?

	Yes	No
a. Another health care provider? ESent_AnotherHCP	1	2
b. A family member or another person involved with your care? ESent_Family	1	2
c. A service or app that can help manage and store your health information? ESent_HealthApp	1	2

D11. In general, how useful is your online medical record for monitoring your health?

UsefulOnlineMedRec

1 Very useful
2 Somewhat useful
3 Not very useful
4 Not at all useful
5 I do not use my online medical record to monitor my health

E: Caregiving

E1. Are you currently caring for or making health care decisions for someone with a **medical, behavioral, disability, or other condition?**

Mark all that apply.

1 Yes, a child/children Caregiving_Child
1 Yes, a spouse/partner Caregiving_Spouse
1 Yes, a parent/parents Caregiving_Parent
1 Yes, another family member Caregiving_AnotherFam
1 Yes, a friend or other non-relative Caregiving_Friend
1 No → **GO TO E11 on page 10** Caregiving_No

CaregivingWho_Cat

E2. Do you provide any of this care professionally as part of a job (for example, as a nurse or professional home health aide)?

Caregiving_Professional

1 Yes
2 No

55346

E3. Please think about the individual for whom you are currently providing the most care.

Please check all conditions for which you have provided care for this person.

Mark all that apply.

- 1 **Cancer** Caregiving_Cancer
- 1 **Alzheimer's, confusion, dementia, forgetfulness** Caregiving_Alzheimers
- 1 **Orthopedic/Musculoskeletal Issues** (examples: back problems, broken bones, arthritis, mobility problems, can't get around, feeble, unsteady, falling) Caregiving_OrthoMusc
- 1 **Mental health/behavioral/substance abuse issues** (examples: mental illness, emotional problems, depression, anxiety, substance/drug/alcohol abuse) Caregiving_MentalHealth
- 1 **Chronic conditions** (examples: high blood pressure/hypertension, diabetes, heart disease, heart attack, lung disease, emphysema, Chronic Obstructive Pulmonary Disease (COPD), Parkinson's) Caregiving_ChronicCond
- 1 **Neurological/developmental Issues** (examples: brain damage or injury, developmental or intellectual disorder, mental retardation, Down syndrome, stroke) Caregiving_NeuroDev
- 1 **Acute conditions** Caregiving_AcuteCond
- 1 **Aging/aging related health issues not listed in the other categories above** Caregiving_Aging
- 1 **Other – Specify** → Caregiving_Other Caregiving_Other_OS
- 1 **Not sure/don't know** Caregiving_NotSure

CaregivingCond_Cat

E4. Think about the individual for whom you are currently providing the most care. About how many hours per week do you spend in an average week providing care?

[| |] Hours spent providing care per week

Caregiving_HoursPerWeek2

E5. Think about the individual for whom you are currently providing the most care. About how long have you been providing care for this person? Your best estimate is fine. Caregiving_HowLong

- 1 Less than 30 days
- 2 1 to 6 months
- 3 7 months to 2 years
- 4 3 to 5 years
- 5 More than 5 years

E6. Think about the individual for whom you are currently providing the most care. Where does your care recipient live currently? Caregiving_Reside

- 1 In your household
- 2 Within 20 minutes of your home
- 3 20 to 59 minutes from your home
- 4 1 to 2 hours from your home
- 5 More than 2 hours away from your home

E7. Think about the individual for whom you are currently providing the most care. Do you help your care recipient with any of the following activities?

Mark all that apply.

- 1 Getting in and out of beds and chairs Caregiving_BedsChairs
- 1 Getting dressed Caregiving_Dressing
- 1 Getting to and from the toilet Caregiving_Toilet
- 1 Dealing with incontinence or diapers Caregiving_Incontinence
- 1 Bathing/Personal care Caregiving_Bathing
- 1 Preparing meals Caregiving_MealPrep
- 1 Feeding him/her Caregiving_Feeding
- 1 Managing finances such as paying bills or filling out insurance claims Caregiving_Finances
- 1 Grocery shopping or other shopping Caregiving_Shopping
- 1 Housework Caregiving_Housework
- 1 Transportation (driving him/her or arranging transportation) Caregiving_Transportation

CaregivingActivities_Cat

E8. Think about the individual for whom you are currently providing the most care. Do you help your care recipient with any of the following activities?

Mark all that apply.

- 1 Performing medical/nursing tasks (for example, giving medicine, changing bandages, managing side effects or symptoms) Caregiving_MedTasks
- 1 Communicating with health care professionals like doctors, nurses, or social workers about his/her care Caregiving_CommunicateHCP
- 1 Arranging for outside services such as nurses, home care aides, Meals on Wheels, or other community services Caregiving_ArrangeSvcs
- 1 Spending time with him/her and providing companionship or emotional support Caregiving_SpendTime

Caregiving_MedAct_Cat

55346

E9. Think about the individual for whom you are currently providing the most care. How many times did you access your care recipient's online medical record in the last 12 months?

Caregiving_AccessMedRec

- 0 None
- 1 1 to 2 times
- 2 3 to 5 times
- 3 6 to 9 times
- 4 10 or more times

E10. The next few questions are about you as a caregiver. Have you ever needed and/or received any of the following care support services?

	Did not need	Received	Needed and not received
a. Training on how to perform medical/nursing tasks (for example, giving medicine, changing bandages, managing side effects or symptoms)................ Caregiver_MedTrain	1	2	3
b. Help in getting access to services such as nurses, home care aides, Meals on Wheels or other community services......................... Caregiver_AccessHelp	1	2	3
c. Short-term or long-term breaks for caregivers (respite care)............. Caregiver_RespiteCare	1	2	3
d. Support groups for caregivers.......... Caregiver_SupportGroup	1	2	3
e. Individual counseling to help cope with giving care....................... Caregiver_Counseling	1	2	3

E11. Medical/nursing tasks include activities such as administering medicine, changing bandages, or managing side effects or symptoms.

If you had to learn how to do a medical/nursing task to assist someone, how would you prefer to learn?

Mark all that apply.

- 1 In-person instruction CaregiverTraining_InPerson
- 1 Hotline or 24-hr phone number to call with questions CaregiverTraining_Hotline
- 1 Reading material: pamphlet or book CaregiverTraining_ReadingMat
- 1 Online video instruction CaregiverTraining_OnlineVideo
- 1 Virtual: have a qualified person provide me with live instruction over the internet CaregiverTraining_Virtual

CaregiverTraining_Cat

F: Palliative Care

F1. How would you describe your level of knowledge about palliative care?

KnowledgePalliativeCare

- 1 I've never heard of it → **GO TO G1 on the next page**
- 2 I know a little bit about palliative care
- 3 I know what palliative care is and could explain it to someone else

F2 To me, the goal of palliative care is to…

	Strongly agree	Somewhat agree	Somewhat disagree	Strongly disagree	Don't know
a. Help friends and family to cope with a patient's illness.................................. PCGoal_HelpFamCope	1	2	3	4	5
b. Offer social and emotional support.................................. PCGoal_SocEmotSupport	1	2	3	4	5
c. Manage pain and other physical symptoms................ PCGoal_ManageSymptoms	1	2	3	4	5
d. Give patients more time at the end of life........................ PCGoal_MoreTime	1	2	3	4	5

55346

F3. Imagine you had a strong need to get information about palliative care. Where would you go first to get information?

PCStrongNeedInfo

Mark only one.

- 1 Printed materials (for example, newspapers, magazines)
- 2 Health care provider (doctor, nurse, social worker)
- 3 Conversations with people you trust (friends, relatives, or co-workers)
- 4 Internet (Google or another search engine, WebMD or another medical website)
- 5 Social Media (Facebook, Instagram, Twitter)

PCStrongNeedInfo_IMP

F4. Imagine you had a strong need to get information about palliative care. Which of the following would you most trust as a source of information about palliative care?

PCTrustInfo

Mark only one.

- 1 Printed materials (for example, newspapers, magazines)
- 2 Health care provider (doctor, nurse, social worker)
- 3 Conversations with people you trust (friends, relatives, or co-workers)
- 4 Internet (Google or another search engine, WebMD or another medical website)
- 5 Social Media (Facebook, Instagram, Twitter)

PCTrustInfo_IMP

F5. How much do you agree or disagree with the following statements about palliative care?

	Strongly agree	Somewhat agree	Somewhat disagree	Strongly disagree	Don't know
a. Accepting palliative care means giving up.................. PCMeansGivingUp	1	2	3	4	5
b. It is a doctor's obligation to inform all patients with cancer about the option of palliative care.................................... PCObligatedToInform	1	2	3	4	5
c. If you accept palliative care, you must stop other treatments........................... PCStopTreatments	1	2	3	4	5
d. Palliative care is the same as hospice care.................... PCHospiceCare	1	2	3	4	5
e. When I think of "palliative care," I automatically think of death............................... PCThinkDeath	1	2	3	4	5

G: Your Overall Health

G1. In general, would you say your health is...

GeneralHealth

- 1 Excellent,
- 2 Very good,
- 3 Good,
- 4 Fair, or
- 5 Poor?

G2. Overall, how confident are you about your ability to take good care of your health?

OwnAbilityTakeCareHealth

- 1 Completely confident
- 2 Very confident
- 3 Somewhat confident
- 4 A little confident
- 5 Not confident at all

55346

G3. Has a doctor or other health professional ever told you that you had any of the following medical conditions:

	Yes	No
a. Diabetes or high blood sugar? MedConditions_Diabetes	1	2
b. High blood pressure or hypertension? MedConditions_HighBP	1	2
c. A heart condition such as heart attack, angina, or congestive heart failure? MedConditions_HeartCondition	1	2
d. Chronic lung disease, asthma, emphysema, or chronic bronchitis? MedConditions_LungDisease	1	2
e. Arthritis or rheumatism? MedConditions_Arthritis	1	2
f. Depression or anxiety disorder? MedConditions_Depression	1	2

G4. About how tall are you without shoes?

☐ Feet ***and*** ☐☐ Inches

Height_Feet; Height_Inches

G5. About how much do you weigh, in pounds, without shoes?

☐☐☐ Pounds Weight

G6. Over the past 2 weeks, how often have you been bothered by any of the following problems?

	Nearly every day	More than half the days	Several days	Not at all
a. Little interest or pleasure in doing things LittleInterest	1	2	3	4
b. Feeling down, depressed, or hopeless Hopeless	1	2	3	4
c. Feeling nervous, anxious, or on edge Nervous	1	2	3	4
d. Not being able to stop or control worrying Worrying	1	2	3	4

G7. Now we are going to ask you about other people in your life. Please respond to each item by marking one box per row.

	Never	Rarely	Sometimes	Often	Always
a. Is there anyone you can count on to provide you with emotional support when you need it – such as talking over problems or helping you make difficult decisions? EmotionalSupport2	1	2	3	4	5
b. Do you have friends or family members that you talk to about your health? TalkHealthFriends2	1	2	3	4	5
c. Do you have someone to prepare your meals if you are unable to do it yourself? HelpPreparingMeals	1	2	3	4	5
d. Do you have someone to take you to the doctor if you need it? HelpTransportDoctor	1	2	3	4	5
e. Do you have someone to help with your daily chores if you are sick? HelpDailyChores2	1	2	3	4	5
f. Do you have someone to run errands if you need it? HelpRunErrands	1	2	3	4	5

G8. Are you deaf or do you have serious difficulty hearing? Deaf

1 Yes
2 No

55346

H: Health and Nutrition

H1. How much do you think that each of the following can influence whether or not a person will develop cancer?

	A lot	A little	Not at all	Don't know
a. Being overweight or obese....... InfluenceCancer_Obesity	1	2	3	4
b. Eating healthy food.................. InfluenceCancer_EatingHealthy	1	2	3	4
c. Exercising regularly.................. InfluenceCancer_RegExercise	1	2	3	4

H2. Think about <u>the last time</u> you ordered food in a fast food or sit down restaurant, did you notice calorie information listed next to the food on the menu or menu board? NoticeCalorieInfoOnMenu

1 Yes

2 No → **GO TO H5 in the next column**

H3. Thinking about <u>the last time</u> you noticed calorie information on the menu or menu board, how easy or difficult to <u>understand</u> was the calorie information? UnderstandCalorieInfo

1 Very easy

2 Somewhat easy

3 Somewhat difficult

4 Very difficult

H4. Thinking about <u>the last time</u> you noticed calorie information on the menu or menu board, how did the calorie information change what you were thinking of ordering?

	Yes	No
a. I ordered something with fewer calories...... CalorieInfo_FewerCalories	1	2
b. I ordered something with more calories....... CalorieInfo_MoreCalories	1	2
c. I ordered fewer items.................................. CalorieInfo_FewerItems	1	2
d. I ordered smaller sizes................................ CalorieInfo_SmallerSizes	1	2
e. I ordered more items.................................. CalorieInfo_MoreItems	1	2
f. I ordered larger sizes.................................. CalorieInfo_LargerSizes	1	2

H5. About how many calories do you think a man/woman of your age and physical activity needs to consume a day to maintain your current weight? AverageCaloriesPerDay

☐☐☐☐ Calories

8 Don't know AverageCaloriesPerDay_DK

H6. These are examples of one drink of alcohol:

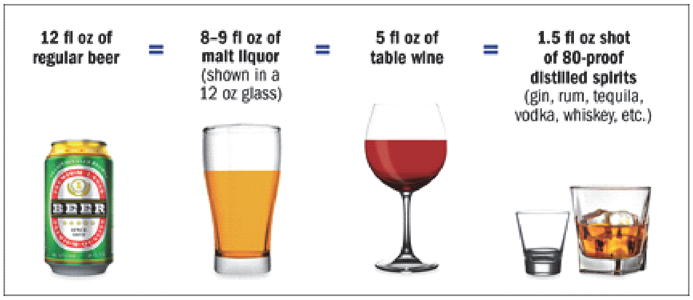

During the past 30 days, <u>how many days per week</u> did you have at least one drink of any alcoholic beverage? DrinkDaysPerWeek

☐ Days per week

→ **(If 0 then go to I1 on the next page)**

H7. During the past 30 days, <u>on the days when you drank</u>, about how many drinks did you drink on average? DrinksPerDay

☐☐ Average drinks <u>per day</u>

55346

I: Physical Activity, Exercise, and UV Exposure

I1. In a typical week, how many days do you do any physical activity or exercise of at least moderate intensity, such as brisk walking, bicycling at a regular pace, and swimming at a regular pace (do not include weightlifting)?

TimesModerateExercise

- 0 None → **GO TO I3 below**
- 1 1 day per week
- 2 2 days per week
- 3 3 days per week
- 4 4 days per week
- 5 5 days per week
- 6 6 days per week
- 7 7 days per week

I2. On the days that you do any physical activity or exercise of at least moderate intensity, how long do you typically do these activities?

[| |] **Minutes per day**

HowLongModerateExerciseMinutes

I3. In a typical week, outside of your job or work around the house, how many days do you do leisure-time physical activities specifically designed to strengthen your muscles such as lifting weights or circuit training (do not include cardio exercise such as walking, biking, or swimming)?

TimesStrengthTraining

- 0 None
- 1 1 day per week
- 2 2 days per week
- 3 3 days per week
- 4 4 days per week
- 5 5 days per week
- 6 6 days per week
- 7 7 days per week

I4. During the past 7 days, how much time did you spend sitting on a typical day at home or at work? This may include time spent sitting at a desk, visiting friends, reading, driving or riding in a car, or sitting or lying down to watch television.

AverageTimeSitting

[|] **Hours per day**

I5. In the past 12 months, how many times have you used a tanning bed or booth?

TimesUsedTanningBed

[| |] Times

I6. On warm sunny days, how often do you spend time in the sun in order to get a tan?

SpendTimeInSunTanning

- 1 Often
- 2 Sometimes
- 3 Rarely
- 4 Never
- 5 Don't go out on sunny days

I7. During the past 12 months, has a physician or other health care provider talked to you about reducing your exposure to the sun or indoor tanning devices such as sunlamps, sunbeds, or tanning booths to reduce the risk for skin cancer?

HCPAdvisedLimitingSun

- 1 Yes
- 2 No

I8. After several months of not being in the sun very much, if you went out in the sun for an hour without sunscreen, a hat or protective clothing, which one of these best describes what would happen to your skin?

SunEffectAfter1Hour

- 1 Get a severe sunburn with blisters
- 2 Have a moderate sunburn with peeling
- 3 Burn mildly with some or no tanning
- 4 Turn darker without sunburn
- 5 Nothing would happen to my skin

55346

J: Tobacco Products

J1. Have you smoked at least 100 cigarettes in your entire life? Smoke100

- 1 Yes
- 2 No → **GO TO J3 below**

J2. How often do you now smoke cigarettes? SmokeNow

- 1 Every day
- 2 Some days
- 3 Not at all

J3. New types of cigarettes are now available called electronic cigarettes or e-cigarettes (also known as vape-pens, hookah pens, e-hookahs, or e-vaporizers). These products deliver nicotine through a vapor. Compared to smoking cigarettes, would you say that electronic cigarettes are… ElecCigLessHarm

- 1 Much less harmful,
- 2 Less harmful,
- 3 Just as harmful,
- 4 More harmful,
- 5 Much more harmful, or
- 7 I don't know

J4. Have you ever used an e-cigarette, even one or two times? UsedECigEver

- 1 Yes
- 2 No → **GO TO J6 in the next column**

J5. Do you now use an e-cigarette every day, some days, or not at all? UseECigNow

- 1 Every day
- 2 Some days
- 3 Not at all

J6. In the past 6 months, have you seen messages in newspapers or on television that say that a Federal Court has ordered tobacco companies to make statements about the dangers of smoking cigarettes? SeenFederalCourtTobaccoMessages

- 1 Yes
- 2 No → **GO TO K1 on the next page**

J7. Which of the following messages have you seen?

Mark <u>all that apply</u>.

- 1 That a Federal Court has ordered tobacco companies to make statements about the <u>health effects of smoking</u>. TobaccoMessages_HESmoking
- 1 That a Federal Court has ordered tobacco companies to make statements about the <u>health effects of secondhand smoke</u>. TobaccoMessages_HESecondhand
- 1 That a Federal Court has ordered tobacco companies to make statements about <u>the addictiveness of smoking and nicotine</u>. TobaccoMessages_Addictiveness
- 1 That a Federal Court has ordered tobacco companies to make statements about <u>how cigarettes are designed to enhance the delivery of nicotine.</u> TobaccoMessages_EnhanceDelivery
- 1 That a Federal Court has ordered tobacco companies to make statements about <u>low tar and light cigarettes being just as harmful as regular cigarettes</u>.

TobaccoMessages_LowTarLight

TobaccoMessages_Cat

55346

K: Screening for Cancer

K1. Are you male or female? GenderC

1 Male → **GO TO K4 below**
2 Female

K2. How long ago did you have your most recent Pap test to check for cervical cancer? WhenPapTest

1 A year ago or less
2 More than 1, up to 2 years ago
3 More than 2, up to 3 years ago
4 More than 3, up to 5 years ago
5 More than 5 years ago
6 I have never had a Pap test

K3. When did you have your most recent mammogram to check for breast cancer, if ever? WhenMammogram

1 A year ago or less
2 More than 1, up to 2 years ago
3 More than 2, up to 3 years ago
4 More than 3, up to 5 years ago
5 More than 5 years ago
6 I have never had a mammogram

K4. There are a few different tests to check for colon cancer. These tests include:

A **colonoscopy** – For this test, a tube is inserted into your rectum and you are given medication that may make you feel sleepy. After the procedure, you need someone to drive you home.

A **sigmoidoscopy** – For this test, you are awake when the tube is inserted into your rectum. After the test you can drive yourself home.

A **stool blood test** – For this test, you collect a stool sample at home, and then provide it to a doctor or lab for testing

Have you ever had one of these tests to check for colon cancer? EverTestedColonCa

1 Yes
2 No

L: HPV Awareness

L1. Have you ever heard of HPV? HPV stands for Human Papillomavirus. It is not HIV, HSV, or herpes. HeardHPV

1 Yes
2 No → **GO TO L3 below**

L2. Do you think HPV can cause…

	Yes	No	Not sure
a. Cervical Cancer?............................. HPVCauseCancer_Cervical	1	2	3
b. Penile Cancer?................................ HPVCauseCancer_Penile	1	2	3
c. Anal Cancer?.................................. HPVCauseCancer_Anal	1	2	3
d. Oral Cancer?.................................. HPVCauseCancer_Oral	1	2	3

L3. A vaccine to prevent HPV infection is available and is called the HPV shot, cervical cancer vaccine, GARDASIL®, or Cervarix®.

Before today, have you ever heard of the cervical cancer vaccine or HPV shot? HeardHPVVaccine2

1 Yes
2 No

L4. Including yourself, is anyone in your immediate family between the ages of 9 and 27 years old? FamBetween9and27

1 Yes
2 No → **GO TO M1 on the next page**

L5. In the last 12 months, has a doctor or health care professional recommended that you or someone in your immediate family get an HPV shot or vaccine? RecommendHPVShot

1 Yes
2 No
3 I don't know

55346

M: Your Cancer History

M1. Have you ever been diagnosed as having cancer? EverHadCancer

- 1 Yes
- 2 No → **GO TO N1 on the next page**

M2. What type of cancer did you have?

Mark all that apply.

- 1 Bladder cancer CaBladder
- 1 Bone cancer CaBone
- 1 Breast cancer CaBreast
- 1 Cervical cancer (cancer of the cervix) CaCervical
- 1 Colon cancer CaColon
- 1 Endometrial cancer (cancer of the uterus) CaEndometrial
- 1 Head and neck cancer CaHeadNeck
- 1 Leukemia/Blood cancer CaLeukemia
- 1 Liver cancer CaLiver
- 1 Lung cancer CaLung
- 1 Lymphoma (Hodgkin's) CaHodgkins
- 1 Lymphoma (Non-Hodgkin's) CaNonHodgkin
- 1 Melanoma CaMelanoma
- 1 Oral cancer CaOral
- 1 Ovarian cancer CaOvarian
- 1 Pancreatic cancer CaPancreatic
- 1 Pharyngeal (throat) cancer CaPharyngeal
- 1 Prostate cancer CaProstate
- 1 Rectal cancer CaRectal
- 1 Renal (kidney) cancer CaRenal
- 1 Skin cancer, non-melanoma CaSkin
- 1 Stomach cancer CaStomach
- 1 Other – Specify → CaOther CaOther_OS

Cancer_Cat

M3. At what age were you first told that you had cancer? WhenDiagnosedCancer

[| |] Age

M4. How worried are you about getting cancer again? FreqWorryCancerAgain

- 1 Not at all
- 2 Slightly
- 3 Somewhat
- 4 Moderately
- 5 Extremely

M5. How easy is it for you to imagine yourself developing cancer again in the future? ImagineCancerAgain

- 1 Extremely difficult
- 2 Somewhat difficult
- 3 Neither difficult nor easy
- 4 Somewhat easy
- 5 Extremely easy

If you have been diagnosed as having cancer then please go to N3 on the next page.

55346

N: Beliefs About Cancer

Think about cancer in general when answering the questions in this section.

N1. How worried are you about getting cancer? FreqWorryCancer

- 1 Not at all
- 2 Slightly
- 3 Somewhat
- 4 Moderately
- 5 Extremely

N2. How easy is it for you to imagine yourself developing cancer in the future? ImagineCancer

- 1 Extremely difficult
- 2 Somewhat difficult
- 3 Neither difficult nor easy
- 4 Somewhat easy
- 5 Extremely easy

N3. How much do you agree or disagree with each of the following statements?

	Strongly agree	Somewhat agree	Somewhat disagree	Strongly disagree
a. It seems like everything causes cancer.. EverythingCauseCancer	1	2	3	4
b. There's not much you can do to lower your chances of getting cancer.. PreventNotPossible	1	2	3	4
c. There are so many different recommendations about preventing cancer, it's hard to know which ones to follow........... TooManyRecommendations	1	2	3	4

N4. The following questions ask about your knowledge about cancer in your family. By family we mean your first and second degree biological relatives; that is, your grandparents, parents, brothers and sisters, children, aunts and uncles, nieces and nephews.

A family cancer history is a record of the cancers in your family. This includes knowing if you have no history of cancers in your family.

How well do you know your family's cancer history? FamiliarFamilyCancer

- 1 Very well
- 2 Well
- 3 Somewhat
- 4 A little
- 5 Not at all

N5. Have you ever had a discussion about your family cancer history with any of the following people? If there is no cancer in your family, and you have discussed this, please include that.

Mark all that apply.

- 1 Your biological mother FamilyCancer_Mother
- 1 Your biological father FamilyCancer_Father
- 1 Your biological sister(s) FamilyCancer_Sister
- 1 Your biological brother(s) FamilyCancer_Brother
- 1 Your biological children FamilyCancer_Children
- 1 Other biological family members FamilyCancer_OthFam
- 1 A health care provider FamilyCancer_HCP
- 1 I have not had discussions with any of these people FamilyCancer_None

FamilyCancer_Cat

55346

N6. Many medical forms ask you questions about cancer in your family. How confident are you that you could complete a summary of your family cancer history on a medical form? ConfidentFamilyHistory

- 1 Completely confident
- 2 Very confident
- 3 Somewhat confident
- 4 A little confident
- 5 Not confident at all

N7. Have any of your family members ever had cancer? FamilyEverHadCancer

- 1 Yes
- 2 No
- 4 Not sure

O: You and Your Household

O1. What is your age? Age

[ ][ ][ ] Years old

O2. What is your current occupational status? OccupationStatus

Mark only one.

- 1 Employed Employed
- 2 Unemployed UnEmployed
- 3 Homemaker Homemaker
- 4 Student Student
- 5 Retired Retired
- 6 Disabled Disabled
- 91 Other – Specify → OccupationStatus_OS

OtherOcc
MultiOcc

O3. Have you ever served on active duty in the U.S. Armed Forces, military Reserves or National Guard? Active duty does not include training in the Reserves or National Guard, but DOES include activation, for example, for the Persian Gulf War. ActiveDutyArmedForces

- 1 Yes, now on active duty
- 2 Yes, on active duty in the last 12 months but not now
- 3 Yes, on active duty in the past, but not in the last 12 months
- 4 No, training for Reserves or National Guard only
- 5 No, never served in the military

GO TO O5 below

O4. In the past 12 months, have you received some or all of your health care from a VA hospital or clinic? ReceivedCareVA

- 1 Yes, all of my health care
- 2 Yes, some of my health care
- 3 No, no VA health care received

O5. What is your marital status? MaritalStatus

Mark only one.

- 1 Married
- 2 Living as married
- 3 Divorced
- 4 Widowed
- 5 Separated
- 6 Single, never been married

O6. What is the highest grade or level of schooling you completed? Education

- 1 Less than 8 years
- 2 8 through 11 years
- 3 12 years or completed high school
- 4 Post high school training other than college (vocational or technical)
- 5 Some college
- 6 College graduate
- 7 Postgraduate

55346

O7. Were you born in the United States?

BornInUSA

1 Yes → **GO TO O9 below**

2 No

O8. In what year did you come to live in the United States? YearCameToUSA

				Year

O9. How well do you speak English?

SpeakEnglish

1 Very well

2 Well

3 Not well

4 Not at all

O10. Are you of Hispanic, Latino/a, or Spanish origin? One or more categories may be selected.

Mark <u>all that apply</u>.

1 No, not of Hispanic, Latino/a, or Spanish origin NotHisp

1 Yes, Mexican, Mexican American, Chicano/a Mexican

1 Yes, Puerto Rican PuertoRican

1 Yes, Cuban Cuban

1 Yes, another Hispanic, Latino/a, or Spanish origin OtherHisp

Hisp_Cat

O11. What is your race? One or more categories may be selected.

Mark <u>all that apply</u>.

1 White White

1 Black or African American Black

1 American Indian or Alaska Native AmerInd

1 Asian Indian AsInd

1 Chinese Chinese

1 Filipino Filipino

1 Japanese Japanese

1 Korean Korean

1 Vietnamese Vietnamese

1 Other Asian OthAsian

1 Native Hawaiian Hawaiian

1 Guamanian or Chamorro Guamanian

1 Samoan Samoan

1 Other Pacific Islander OthPacIsl

Race_Cat2

O12. Do you think of yourself as…

SexualOrientation

1 Heterosexual, or straight

2 Homosexual, or gay or lesbian

3 Bisexual

91 Something else – Specify

SexualOrientation_OS

SexualOrientation_I

O13. <u>Including yourself</u>, how many people live in your household? TotalHousehold

		Number of people

55346

O14. Starting with yourself, please mark the sex, and write in the age and month of birth for each adult 18 years of age or older living at this address.

	Sex	Age	Month Born (01-12)
**SELF**	1 Male 2 Female SelfGender	SelfAge	SelfMOB
Adult 2	1 Male 2 Female HHAdultGender2	HHAdultAge2	HHAdultMOB2
Adult 3	1 Male 2 Female HHAdultGender3	HHAdultAge3	HHAdultMOB3
Adult 4	1 Male 2 Female HHAdultGender4	HHAdultAge4	HHAdultMOB4
Adult 5	1 Male 2 Female HHAdultGender5	HHAdultAge5	HHAdultMOB5

O15. How many children under the age of 18 live in your household? ChildrenInHH

[ ][ ] Number of children under 18

O16. Do you currently rent or own your home? RentOrOwn

1 Own
2 Rent
3 Occupied without paying monetary rent

O17. Thinking about members of your family living in this household, what is your combined annual income, meaning the total pre-tax income from all sources earned in the past year? IncomeRanges

1 $0 to $9,999
2 $10,000 to $14,999
3 $15,000 to $19,999
4 $20,000 to $34,999
5 $35,000 to $49,999
6 $50,000 to $74,999
7 $75,000 to $99,999
8 $100,000 to $199,999
9 $200,000 or more

O18. About how long did it take you to complete the survey?

Write a number in one box below.

[ ][ ] Minutes [ ][ ] Hours

MailSurveyTimeMin
MailSurveyTimeHrs

O19. At which of the following types of addresses does your household currently receive residential mail?

Mark all that apply.

1 A street address with a house or building number TypeOfAddressA
1 An address with a rural route number TypeOfAddressB
1 A U.S. post office box (P.O. Box) TypeOfAddressC
1 A commercial mailbox establishment (such as Mailboxes R Us, and Mail Boxes Etc.®) TypeOfAddressD

55346

Thank you!

Please return this questionnaire in the postage-paid envelope within 2 weeks.

If you have lost the envelope, mail the completed questionnaire to:

HINTS Study, TC 1046F
Westat
1600 Research Boulevard
Rockville, MD 20850
