# Supplementary material for: Characterizing the US Population by Patterns of Mobile Health Use for Health and Behavioral Tracking: Analysis of the National Cancer Institute's Health Information National Trends Survey Data
Source: J Med Internet Res. 2020 May 14;22(5):e16299. doi: 10.2196/16299 (PMC7256752; doi:10.2196/16299)
Supplement: Multimedia Appendix 3 [file jmir_v22i5e16299_app3.pdf]

Weighted Population Estimates for Sociodemographic and Health-Related Characteristics, HINTS 5, Cycle 1, 2017 and HINTS 5, Cycle 2, 2018 (N=6,789)

Characteristic	Respondents, n (Weighted %)
**Gender**	
Male	2,564 (49.01)
Female	3,697 (50.99)
**Age (years)**	
18-34	773 (22.75)
35-49	1,313 (27.67)
50-64	2,176 (30.25)
≥ 65	2,299 (19.32)
**Race and ethnicity**	
White, non-Hispanic	3,851 (65.23)
Black, non-Hispanic	853 (10.56)
Hispanic	888 (15.87)
Other	512 (8.35)
**Education**	
Less than high school	492 (8.83)
High school graduate	1,247 (22.63)
Technical, vocational, or some college	1,981 (36.40)
College graduate or postgraduate	2,914 (32.14)
**Income in US $**	
<20,000	1,238 (17.72)
20,000-49,999	1,794 (26.19)
50,000-74,999	1,190 (18.57)
≥75,000	2,418 (37.52)
**Geographical area**	
Urban	5,863 (86.07)
Rural	926 (13.93)
**Perceived health status**	
Poor	183 (2.65)
Fair	956 (13.25)
Good	2,347 (34.54)
Very good	2,449 (36.19)
Excellent	780 (13.37)
**Health self-efficacy**	
Not confident at all	80 (1.45)
A little confident	223 (3.50)
Somewhat confident	1,644 (25.40)
Very confident	3,120 (44.99)
Completely confident	1,643 (24.66)
**Health information seeker**	
Yes	5,379 (79.68)
No	1,335 (20.32)

Characteristic	Respondents, n (Weighted %)
**BMI (kg/m^2)**	
Normal (18.5-24.9)	2,034 (32.44)
Overweight (25-29.9)	2,265 (33.68)
Obese (≥30)	2,169 (33.88)
**One or more chronic conditions**	
0	3,170 (57.79)
1	2,046 (26.99)
2+	1,337 (15.22)
